# A randomized, placebo‐controlled trial evaluating effects of lebrikizumab on airway eosinophilic inflammation and remodelling in uncontrolled asthma (CLAVIER)

**DOI:** 10.1111/cea.13731

**Published:** 2020-10-04

**Authors:** Cary D. Austin, Melissa Gonzalez Edick, Ronald E. Ferrando, Margaret Solon, Miriam Baca, Kathryn Mesh, Peter Bradding, Gail M. Gauvreau, Kaharu Sumino, J. Mark FitzGerald, Elliot Israel, Lief Bjermer, Arnaud Bourdin, Joseph R. Arron, David F. Choy, Julie K. Olsson, Francis Abreu, Monet Howard, Kit Wong, Fang Cai, Kun Peng, Wendy S. Putnam, Cécile T.J. Holweg, John G. Matthews, Monica Kraft, Prescott G. Woodruff, the CLAVIER Investigators

**Affiliations:** ^1^ Genentech, Inc. South San Francisco CA USA; ^2^ University of Leicester and Glenfield Hospital Leicester UK; ^3^ McMaster University Hamilton ON Canada; ^4^ Washington University School of Medicine in St. Louis St Louis MO USA; ^5^ University of British Columbia Vancouver BC Canada; ^6^ Brigham and Women’s Hospital Boston MA USA; ^7^ Skåne University Hospital Lund Sweden; ^8^ CHU de Montpellier Montpellier France; ^9^ University of Arizona College of Medicine Tucson AZ USA; ^10^ San Francisco Medical Center University of California San Francisco CA USA; ^11^ Present address: Stemcentrx/AbbVie, Inc. South San Francisco CA USA; ^12^ Present address: 23andMe Mountain View CA USA

## Abstract

**Background:**

The anti‐interleukin 13 (IL‐13) monoclonal antibody lebrikizumab improves lung function in patients with moderate‐to‐severe uncontrolled asthma, but its effects on airway inflammation and remodelling are unknown. CLAVIER was designed to assess lebrikizumab's effect on eosinophilic inflammation and remodelling.

**Objective:**

To report safety and efficacy results from enrolled participants with available data from CLAVIER.

**Methods:**

We performed bronchoscopy on patients with uncontrolled asthma before and after 12 weeks of randomized double‐blinded treatment with lebrikizumab (n = 31) or placebo (n = 33). The pre‐specified primary end‐point was relative change in airway subepithelial eosinophils per mm^2^ of basement membrane (cells/mm^2^). Pre‐specified secondary and exploratory outcomes included change in IL‐13‐associated biomarkers and measures of airway remodelling.

**Results:**

There was a baseline imbalance in tissue eosinophils and high variability between treatment groups. There was no discernible change in adjusted mean subepithelial eosinophils/mm^2^ in response to lebrikizumab (95% CI, −82.5%, 97.5%). As previously observed, FEV_1_ increased after lebrikizumab treatment. Moreover, subepithelial collagen thickness decreased 21.5% after lebrikizumab treatment (95% CI, −32.9%, −10.2%), and fractional exhaled nitric oxide, *CCL26* and *SERPINB2* mRNA expression in bronchial tissues also reduced. Lebrikizumab was well tolerated, with a safety profile consistent with other lebrikizumab asthma studies.

**Conclusions & Clinical Relevance:**

We did not observe reduced tissue eosinophil numbers in association with lebrikizumab treatment. However, in pre‐specified exploratory analyses, lebrikizumab treatment was associated with reduced degree of subepithelial fibrosis, a feature of airway remodelling, as well as improved lung function and reduced key pharmacodynamic biomarkers in bronchial tissues. These results reinforce the importance of IL‐13 in airway pathobiology and suggest that neutralization of IL‐13 may reduce asthmatic airway remodelling.

Clinical Trial Registration: NCT02099656.

## 1 INTRODUCTION

Asthma, a chronic, heterogeneous disorder affecting ≈ 300 million people worldwide,^1^, ^2^ is characterized by variable airflow obstruction, airway inflammation, mucus hypersecretion and tissue remodelling, including subepithelial fibrosis. Patients whose asthma remains uncontrolled despite treatment represent a substantial unmet clinical need and are at risk of acute disease worsening.^3^, ^4^ Guideline‐based standard‐of‐care therapy includes inhaled corticosteroids (ICS) plus a second controller medication.^4^

Airway eosinophilic inflammation is a key feature of asthma driven by type 2 (T2) inflammation.^5^ Eosinophil counts in the blood and different airway compartments can be discordant.^6^, ^7^, ^8^ ICS treatment can decrease airway mucosal eosinophils, although eosinophilic airway inflammation persists in some patients.^7^, ^9^

Interleukin (IL)‐13 is a pleiotropic cytokine thought to play a key role in T2‐driven inflammation, including eosinophilic inflammation in severe asthma,^10^ has been implicated in promoting eosinophil survival, activation and recruitment,^11^, ^12^, ^13^ and may also mediate features of airway remodelling relevant to asthma, such as subepithelial fibrosis.^14^, ^15^ Lebrikizumab is a humanized monoclonal antibody that binds soluble IL‐13 to block downstream signalling.^16^, ^17^ Lebrikizumab treatment is associated with increased peripheral blood eosinophils in some patients with asthma, which may have been due to reduced eosinophil trafficking to tissue.^16^, ^18^, ^19^ In phase 2 studies, lebrikizumab reduced the number of exacerbations and improved lung function in patients with moderate‐to‐severe uncontrolled asthma, particularly in those with higher levels of T2 biomarkers such as periostin, blood eosinophils and fractional exhaled nitric oxide (FeNO).^16^, ^20^, ^21^ However, replicate phase 3 trials in adult patients with uncontrolled asthma only partially supported these findings. Lebrikizumab significantly reduced the rate of asthma exacerbations over 52 weeks in biomarker‐high patients (defined as periostin ≥50 ng/mL or blood eosinophils ≥300 cells/μL) in LAVOLTA I (NCT01867125), but this effect was inconsistently observed in LAVOLTA II (NCT01868061).^18^ Nevertheless, both phase 3 trials showed improvements in forced expiratory volume in 1 second (FEV_1_).^18^

CLAVIER (NCT02099656) was a phase 2 bronchoscopy trial that investigated the effects of lebrikizumab on airway inflammation and remodelling in patients with uncontrolled asthma. Based on the mixed efficacy results of the LAVOLTA studies, the lebrikizumab asthma programme was terminated by the sponsor; therefore, CLAVIER drug dosing was terminated and enrolment closed before the planned sample size was achieved. All enrolled patients were invited to complete the study, and here, results from enrolled participants with available data are reported.

## 2 METHODS

### 2.1 Study design

**Figure 1:**
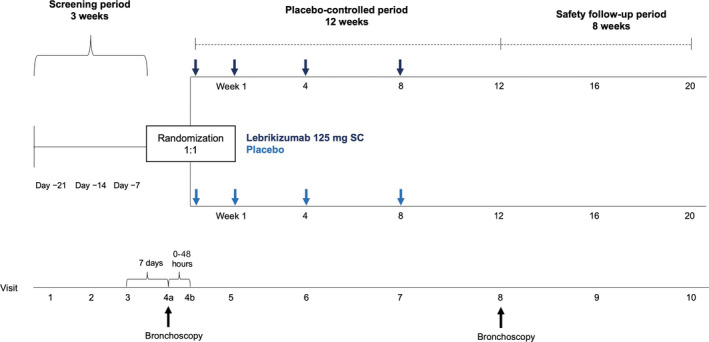
Trial design, with lebrikizumab 125 mg or placebo administered subcutaneously on day 1, day 8, week 4 and week 8 during the 12‐week placebo‐controlled period. SC, subcutaneous

CLAVIER was a phase 2, multi‐centre, randomized, double‐blind, placebo‐controlled clinical trial that incorporated research bronchoscopy. The study consisted of a 3‐week screening period, 12‐week placebo‐controlled treatment period, and 8‐week safety follow‐up period with a planned sample size of 80 patients (Figure 1; Table S1; see Supplement for additional details). Following written informed consent, patients were screened, and bronchoscopy was performed at visit 4a to collect baseline samples. Patients were randomized 1:1 to receive lebrikizumab or placebo stratified by baseline serum periostin level (<50 or ≥50 ng/mL), baseline asthma medications (total daily dose ≥1000 μg fluticasone propionate dry powder inhaler (DPI) or equivalent plus long‐acting beta agonists [LABA; yes, no], and nasosorption/sputum induction substudy participation [yes, no]).

### 2.2 Patients

Eligible patients were 18‐75 years old with a clinical diagnosis of asthma ≥12 months prior to visit 1, documented bronchodilator reversibility (≥12% relative improvement) within 12 months prior to or during screening, and prebronchodilator FEV_1_ of 40%–80% predicted at both screening visits 2 and 3. Patients were receiving total daily dose of 500‐2000 μg fluticasone propionate DPI or equivalent and were on an eligible second controller medication for ≥6 months prior to visit 1, with no changes within 4 weeks prior to visit 1. Eligible second asthma controller medications were LABAs, leukotriene receptor antagonists, long‐acting muscarinic antagonists or theophylline. Doses for ICS and second controllers needed to remain stable throughout the study, except for theophylline which could be adjusted based on blood levels.

Uncontrolled asthma during screening was defined as a five‐item Asthma Control Questionnaire score of ≥ 1.5 and at least one of the following: daytime symptoms >2 d/wk, night‐time awakening ≥ 1 night/wk, rescue medication use on ≥2 d/wk and/or interference with normal daily activities.^22^ Patients had to have documented absence of other clinically significant lung disease and demonstrate adherence to controller medication during screening. Key exclusion criteria are listed in the Supplement.

All patients provided written informed consent prior to study participation. The study was conducted in accordance with the Declaration of Helsinki and the International Conference on Harmonisation Guidance for Good Clinical Practice. Independent ethics committee approval was obtained at all participating centres. An internal data monitoring committee reviewed safety data regularly throughout the trial.

### 2.3 Outcomes

The pre‐specified primary stereologically assessed efficacy end‐point was the placebo‐corrected adjusted mean relative (per cent) change in the number of airway subepithelial (basal lamina plus submucosa) eosinophils per mm^2^ of basement membrane from baseline to week 12. The placebo‐corrected adjusted mean change was defined as the difference in adjusted mean changes between the lebrikizumab and placebo groups.

Pre‐specified secondary stereologically assessed efficacy end‐points, pharmacodynamics and exploratory remodelling‐related stereologically assessed end‐points were also evaluated (see Supplement for full list).

### 2.4 Statistics

Due to our interest in exploring multiple parameters and subgroup analyses in this study, no formal hypothesis tests were performed. Results were reported as point estimates and associated 95% CIs, with no adjustment for multiple comparisons. Details of sample size calculations, analysis populations, stratification and adjustment analyses are provided in the Supplement.

## 3 RESULTS

### 3.1 Patients

**Figure 2:**
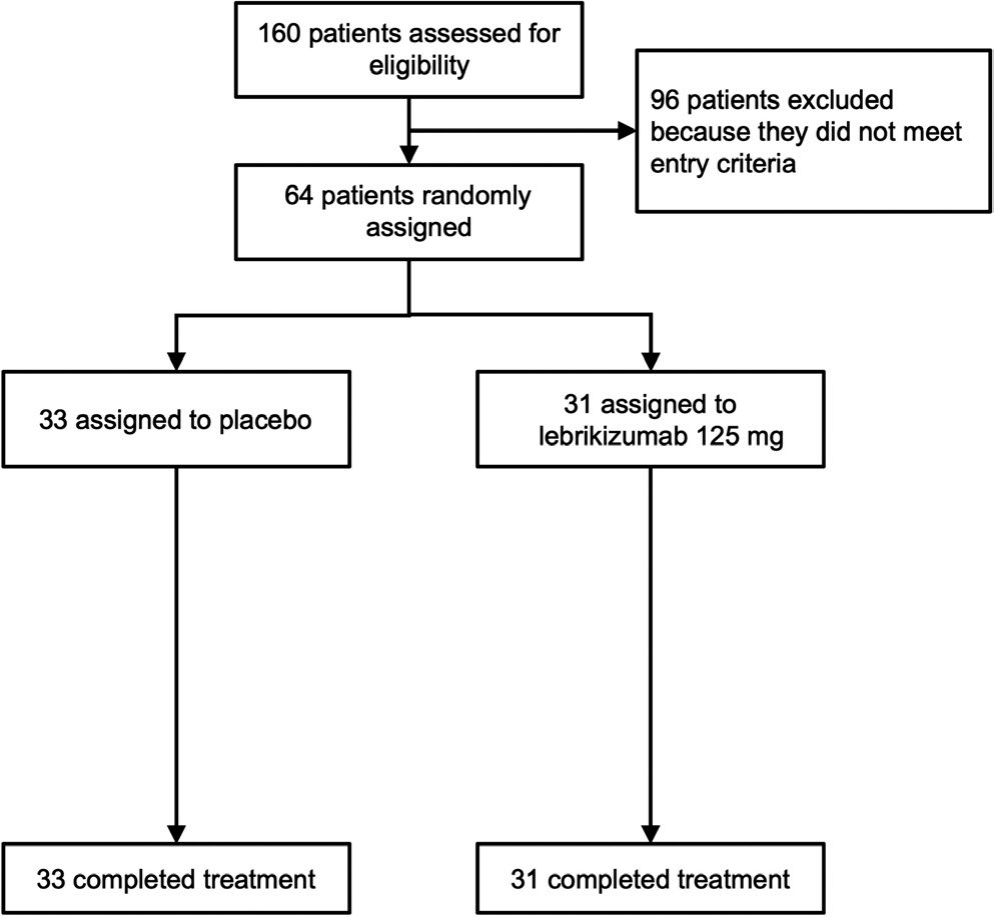
Patient disposition

Enrolment began on 6 November 2014, and the study was concluded on 13 October 2016. Patients already enrolled at the time of programme termination in July 2016 were invited to complete the study. Of the 160 patients screened, 64 were enrolled from 17 centres in five countries (Figure 2). Thirty‐one patients were randomized to lebrikizumab, and 33 to placebo; these patients comprised the intent‐to‐treat (ITT) and safety‐evaluable populations. Thirteen patients were excluded from the ITT/safety‐evaluable population to form the primary analysis population, which comprised 51 patients subject to efficacy analysis: 26 in the lebrikizumab arm and 25 in the placebo arm. ITT‐excluded patients included 10 with failed biopsy quality assurance at baseline and/or week 12 and 3 lacking a week‐12 biopsy.

The primary analysis population was divided into periostin‐high and periostin‐low subgroups, and 41.2% were periostin‐high. Exploratory subgroup analyses were performed by blood eosinophil and FeNO status; 35.3% were eosinophil‐high, and 37.3% were FeNO‐high. Patients treated with placebo vs lebrikizumab within each subgroup were periostin‐low, 13 (43.3%) vs 17 (56.7%); periostin‐high, 12 (57.1%) vs 9 (42.9%); eosinophil‐low, 14 (42.4%) vs 19 (57.6%); eosinophil‐high, 11 (61.1%) vs 7 (38.9%); FeNO‐low, 19 (59.4%) vs 13 (40.6%); and FeNO‐high, 6 (31.6%) vs 13 (68.4%).

All patients completed assessments up to and including week 12. One patient receiving four doses of lebrikizumab discontinued the study at week 16 (safety follow‐up), withdrawing consent due to study termination by the sponsor.

The treatment arms in the ITT population were generally balanced with respect to baseline demographic and disease characteristics, though a racial imbalance was present (Table 1). The mean baseline ICS dose was similar in the lebrikizumab (804 μg/d) and placebo (853 μg/d) arms. Baseline median serum periostin levels were 49.0 and 46.3 ng/mL; median blood eosinophil counts were 230 and 270 cells/μL; and median FeNO was 30.0 and 22.0 ppb in the lebrikizumab and placebo arms, respectively.

**Table 1:** Baseline demographics and characteristics in the ITT population

	Placebo (n = 33)	Lebrikizumab 125 mg (n = 31)
Age, mean (SD), years	43.9 (12.8)	45.9 (12.5)
Sex, n (%)		
Male	19 (57.6)	16 (51.6)
Female	14 (42.4)	15 (48.4)
Race, n (%)		
White	25 (75.8)	15 (48.4)
Black or African American	5 (15.2)	14 (45.2)
Asian	2 (6.1)	0
Body mass index, mean (SD), kg/m^2^	28.3 (5.0)	30.2 (5.0)
Former smoker, n (%)	4 (12.1)	8 (25.8)
Duration of asthma, median (range), years	32 (5‐59)	29 (2‐52)
ICS (fluticasone propionate DPI or equivalent), mean (SD), μg/day	853.0 (468.0)	804.0 (402.3)
Prebronchodilator FEV_1_, mean (SD)		
Absolute, L	2.353 (0.741)	2.121 (0.572)
% predicted	64.7 (12.0)	64.7 (9.3)
Reversibility, mean (SD), %	20.2 (9.3)	22.7 (14.5)
ACQ‐5 score, mean (SD)	2.67 (0.85)	2.52 (0.74)
Serum periostin, median (IQR), ng/mL^a^	46.3 (41.6‐56.7)	49.0 (39.1‐62.6)
FeNO, median (IQR), ppb^a^	22.0 (14.0‐31.0)	30.0 (17.0‐49.0)
Blood eosinophil count, median (IQR), cells/μL^a^	270 (190‐440)	230 (120‐410)
Patients with ≥ 1 exacerbation in previous 12 mo, n (%)	13 (42.0)	9 (30.0)

Abbreviations: ACQ‐5, five‐item Asthma Control Questionnaire; DPI, dry powder inhaler; FeNO, fractional exhaled nitric oxide; FEV_1_, forced expiratory volume in 1 s; ICS, inhaled corticosteroid; IQR, interquartile range; ITT, intent‐to‐treat; LABA, long‐acting beta agonist.

^a^ Measured at screening; baseline is day 21.

### 3.2 Sample quality

The tissues sampled by endobronchial biopsy met quality expectations, with 88% and 60% of biopsies passing criteria for lamina propria and epithelium, respectively (Table S2). Stereological precision was considered acceptable for subepithelial measurements, with coefficient of errors of <0.5 for 95% of subepithelial eosinophil counts and 93% of the associated basement membrane surface area measurements. Coefficient of errors for the epithelial measurements was <0.5 for 32% of epithelial eosinophil counts and 95% of the associated basement membrane surface area measurements.

### 3.3 Primary efficacy end‐point

**Figure 3:**
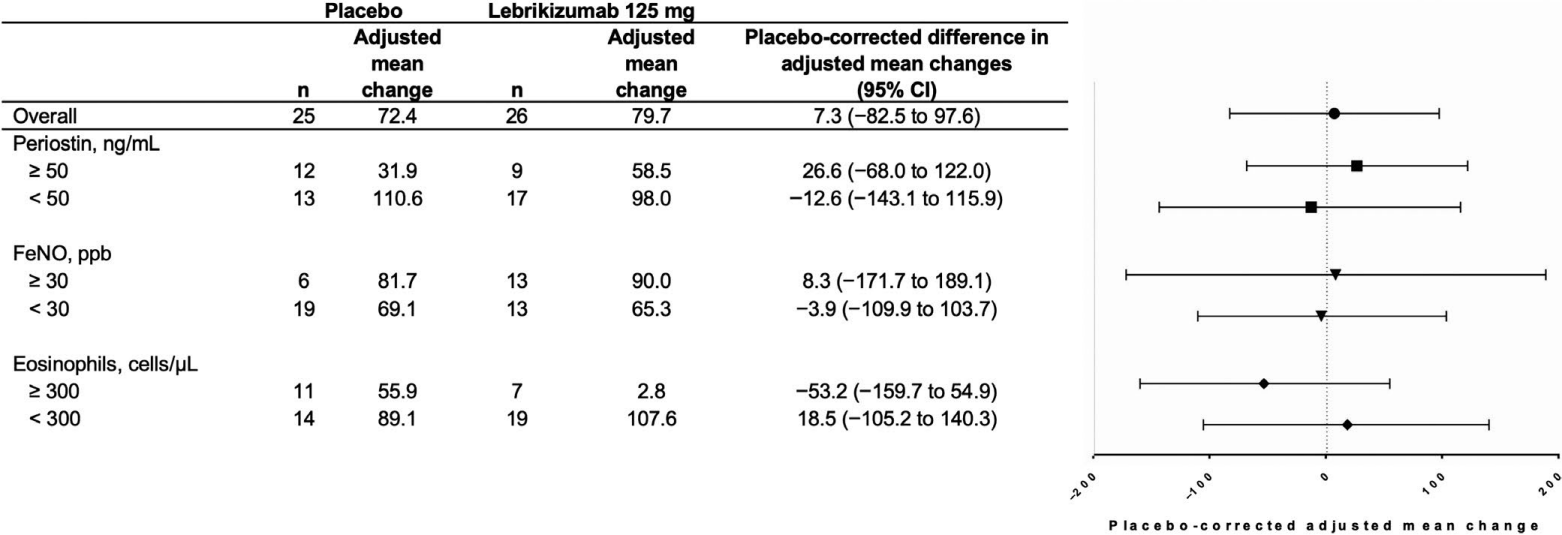
Mean relative (%) changes from baseline in number of airway subepithelial eosinophils per mm^2^ of basement membrane at week 12 in the primary analysis population. Estimates were based on a linear model that used relative change from baseline in airway subepithelial eosinophils as the response variable and included terms for treatment, number of asthma exacerbations within 12 mo of study entry and baseline asthma medications. Relative change was defined as the absolute change from baseline to week 12 divided by the value at baseline. Placebo‐corrected adjusted mean change is the difference in adjusted mean changes between the lebrikizumab and placebo groups. Boxplots of the corresponding unadjusted data are provided in Figure S2

The baseline mean subepithelial eosinophil count per mm^2^ of basement membrane was lower in the lebrikizumab (224 cells/mm^2^ [SD, 228 cells/mm^2^]) vs placebo group (439 cells/mm^2^ [SD, 418 cells/mm^2^]; Table 2). The baseline variability was higher than what was assumed for sample size calculations (418 vs 30 cells/mm^2^). Subepithelial eosinophil counts ranged from 0‐937 and 0‐1639 cells/mm^2^ in the lebrikizumab and placebo groups, respectively. The adjusted mean per cent changes in subepithelial eosinophils at 12 weeks compared with baseline were 79.7% and 72.4% with lebrikizumab and placebo, respectively, resulting in a placebo‐corrected adjusted mean change from baseline of 7.3%, with a wide CI around this estimate (95% CI, −82.5, 97.6). Hence, the aim of 50% reduction in airway eosinophils was not met. No differences in relative change from baseline were observed when comparing lebrikizumab with placebo in any biomarker subgroup (Figure 3; Figure S2).

**Table 2:** Primary efficacy end‐point results. Unadjusted and adjusted relative change in number of airway subepithelial eosinophils per mm^2^ of basement membrane (cells/mm^2^) from baseline to week 12 in the primary analysis population^b^

		Placebo (n = 25)	Lebrikizumab 125 mg (n = 26)
Baseline	Value at visit, mean (SD), cells/mm^2^	439 (418)	224 (228)
Week 12	Value at visit, mean (SD), cells/mm^2^	422 (360)	279 (231)
	Unadjusted change from baseline, mean (SD), %^a^	73.9 (169.4)	75.6 (167.8)
	Adjusted change from baseline, mean (SE), %^b^	72.4 (32.5)	79.7 (32.8)
	95% CI of the difference in adjusted mean changes from baseline	−82.5 to 97.6	

^a^ Mean change from baseline averages patient‐by‐patient changes and is positive in both groups, even though the mean 12‐week value is lower than the mean baseline value in the placebo group. This apparent disconnect is a result of high variability in the data.

^b^ Estimates were based on a linear model that used relative change from baseline in airway subepithelial eosinophils as the response variable and included terms for treatment, number of asthma exacerbations within 12 mo of study entry and baseline asthma medications. Relative change was defined as the absolute change from baseline to week 12 divided by the value at baseline.

### 3.4 Secondary efficacy end‐points

No substantial overall or biomarker subgroup differences were observed in the placebo‐corrected adjusted mean change from baseline in the absolute numbers of subepithelial eosinophils per mm^2^ of basement membrane or per microlitre of lamina propria (Figures S3 and S4).

No absolute or relative changes were observed in epithelial eosinophils per mm^2^ of basement membrane or per microlitre of epithelium; sample sizes in biomarker subgroups for the epithelial eosinophil end‐points were too small for analysis (Table S3).

A clinically meaningful increase in placebo‐corrected mean FEV_1_ was observed in the FeNO‐ and blood eosinophil‐high subgroups, with less pronounced increase in the overall population (Figures S5A,B). A meaningful placebo‐corrected decrease in mean FeNO levels was observed overall and in the periostin‐high subgroup (Figures S5C,D).

### 3.5 Exploratory histological end‐points

To account for the extracellular eosinophil peroxidase (EPO) signal present in some biopsies, a presumed consequence of eosinophil activation and secretory granule secretion, we pursued a non‐stereological method to quantify mean signal intensity per image pixel of subepithelium or epithelium as a post hoc exploratory analysis. As observed in stereological eosinophil counts, pixel‐based EPO quantitation expressed as relative or absolute changes in subepithelium or epithelium revealed substantial variability, with no clear changes attributable to lebrikizumab (Figure S6).

**Figure 4:**
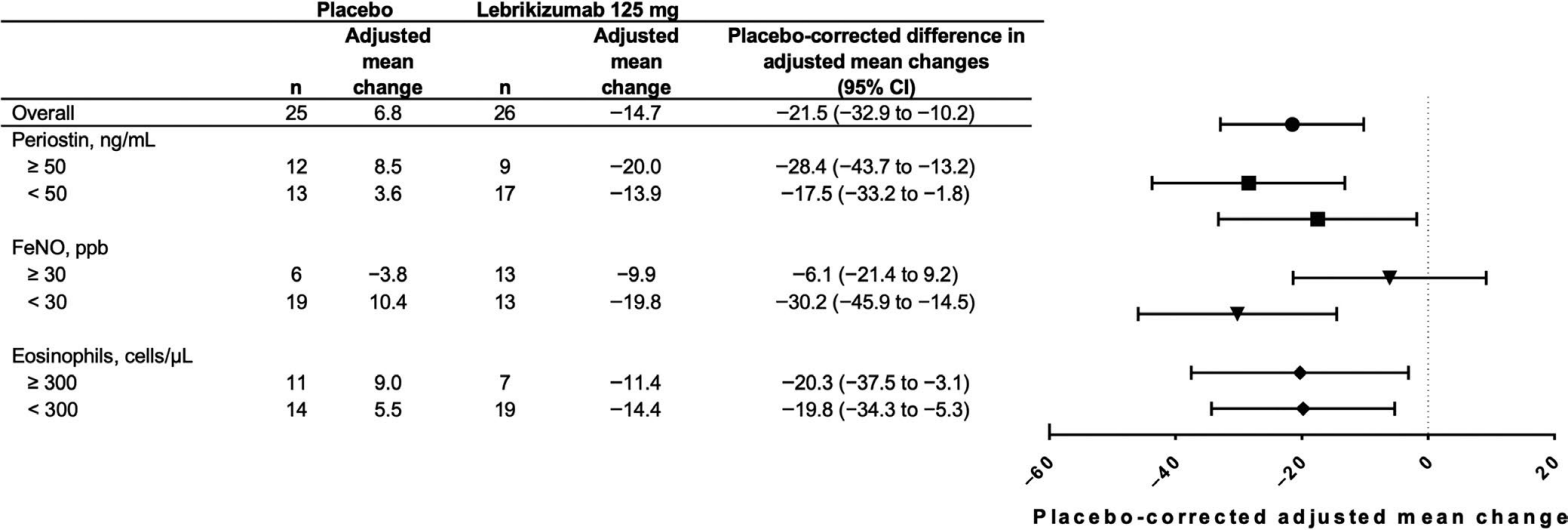
Mean adjusted relative (%) changes from baseline in thickness of subepithelial collagen at week 12 in the primary analysis population. Estimates were based on a linear model that used relative change from baseline in thickness of subepithelial collagen as the response variable and included terms for treatment, number of asthma exacerbations within 12 months of study entry and baseline asthma medications. Relative change was defined as the absolute change from baseline to week 12 divided by the value at baseline. Placebo‐corrected adjusted mean change is the difference in adjusted mean changes between the lebrikizumab and placebo groups. Boxplots of the corresponding unadjusted data are provided in Figure S7

Pre‐specified measures of airway remodelling were evaluated stereologically, including the thickness of the subepithelial collagen layer (degree of “subepithelial fibrosis”), volume of epithelial mucin and goblet cell number. The thickness of subepithelial collagen in the lebrikizumab‐treated group was substantially lower than that in the placebo‐treated group (Figure 4; Figure S7), with a placebo‐corrected adjusted mean change from baseline of −21.5% (95% CI, −32.9%, −10.2%). This decrease was not limited to the T2 biomarker‐high subgroups. The mean change from baseline of epithelial mucin volume per mm^2^ of basement membrane trended lower in the lebrikizumab‐treated group than in the placebo‐treated group, but this difference was not reflected in a similar trend in the number of goblet cells per mm^2^ of basement membrane (Figure S8).

### 3.6 Pharmacodynamics and pharmacokinetics

Circulating chemokine ligand 13 and periostin levels decreased after the first dose of lebrikizumab, but serum total immunoglobulin E levels decreased more gradually (Figure S9). All three biomarker levels remained low throughout the treatment and safety follow‐up period. Lebrikizumab did not change median blood eosinophil counts. None of the pharmacodynamic markers showed obvious changes in the placebo group.

In bronchial tissues, there were trends towards median fold reductions in mRNA expression for *CCL26*, *NOS2*, *SERPINB2, IL13, CLCA1* and *POSTN* in the lebrikizumab arm (n = 13) at week 12 compared with that at baseline in the overall population (n = 17; −0.76 [IQR, 1.91], −0.63 [1.63], −1.01 [2.60], 0.01 [1.18], −1.34 [2.85], and − 0.67 [1.29], respectively; Figure S10).

The mean serum lebrikizumab concentration at week 12 was 13.5 µg/mL (SD, 6.6 µg/mL) and approached steady‐state trough concentrations observed in patients treated at the same dose in other asthma studies.^18^, ^21^, ^23^

### 3.7 Safety

Safety analyses were based on the safety‐evaluable population and included the 12‐week treatment period and 8‐week safety follow‐up. The proportion of patients with at least one AE was similar between the lebrikizumab and placebo arms (69.7% and 67.7%, respectively), and events were mostly of mild or moderate intensity (Table 3). The most common AEs (≥5% incidence) reported across both arms were asthma (16.1% lebrikizumab; 21.2% placebo), dyspnoea (3.2% lebrikizumab; 15.2% placebo), cough (3.2% lebrikizumab; 9.1% placebo) and injection‐site pain (6.2% lebrikizumab; 6.1% placebo). No patients withdrew from the study due to an AE.

**Table 3:** Adverse events^a^

	Placebo (n = 33)	Lebrikizumab 125 mg (n = 31)
Total AEs, n	86	47
SAEs, n	2	6
Deaths, n	0	0
Patients with ≥1 AE, n (%)	23 (69.7)	21 (67.7)
Patients with ≥1 AE assessed as related to study drug by investigator, n (%)	4 (12.1)	2 (6.5)
Patients with ≥1 SAE, n (%)	1 (3.0)	6 (19.4)
Patients with AEs leading to discontinuation from treatment, n (%)	0	0
Patients with AEs of special interest, n (%)		
Injection‐site reaction	2 (6.1)	2 (6.5)
Anaphylaxis per Sampson's criteria^b^	0	0
Infection (broad)^c^	9 (27.3)	9 (29.0)
Malignancy	0	0

Abbreviations: AE, adverse event; SAE, serious adverse event.

^a^ Safety‐evaluable population.

^b^ An independent anaphylaxis adjudication committee evaluated events per Sampson's criteria and assessed relationship to study drug based on a review of blinded data.

^c^ Infections (narrow) were identified based on the Medical Dictionary for Regulatory Activities high‐level group terms of helminthic disorders, mycobacterial infectious disorders and protozoal infectious disorders or high‐level term of listeria infections.

Serious AEs (SAEs) were reported by six patients in the lebrikizumab arm (19.4%; asthma exacerbation [two patients], gastroesophageal reflux disease, leucocytosis, pneumonia, and anaemia) and one patient in the placebo arm (3.0%; pulmonary sepsis and dyspnoea). No SAEs were deemed related to the study drug by the investigators and all resolved without sequelae. Two SAEs were considered related to bronchoscopy: one event of leucocytosis onset on the same day as bronchoscopy and one event of pneumonia onset 2 days after bronchoscopy.

The incidence of AEs of special interest was balanced across both treatment arms. Two patients in each arm (7% lebrikizumab; 6% placebo) experienced an injection‐site reaction. Nine patients in each arm (29.0% lebrikizumab; 27.3% placebo) experienced at least one AE classified as “infections and infestations.” The most frequently reported infections were of the upper respiratory tract, affecting two patients in each treatment arm (6.5% lebrikizumab; 6.1% placebo). No malignancies or anaphylactic, anaphylactoid or hypersensitivity reactions were reported.

No eosinophil‐associated AEs or herpes infections were reported. There were no treatment‐emergent elevations in peripheral blood eosinophils of grade ≥2 (>1500 cells/μL).

The baseline prevalence of ATAs was 1.6% (one of 62 patients). Post‐baseline prevalence of ATAs was 6.9% (two of 29 patients) in patients randomized to lebrikizumab treatment. The two ATA‐positive patients developed low ATA responses, and there was no apparent impact on safety and drug exposure.

## 4 DISCUSSION

CLAVIER was designed primarily to evaluate the effect of lebrikizumab on airway eosinophilic inflammation in patients with moderate‐to‐severe uncontrolled asthma. Research bronchoscopy was employed, providing a valuable opportunity to study the effect of lebrikizumab on airway remodelling. We found no difference in change in tissue eosinophil counts in the lebrikizumab‐ and placebo‐treated groups, although this finding is limited by significant variability, insufficient sample size and a baseline imbalance between groups. However, as part of pre‐specified secondary analyses, lebrikizumab reduced subepithelial fibrosis, a novel finding suggesting a role of IL‐13 in a fundamental aspect of airway remodelling in human asthma. In addition, lebrikizumab improved lung function and reduced T2 biomarkers, suggesting that this dosing regimen had the intended pharmacological and physiological effects.

A wide range of median and variance estimates in tissue eosinophil numbers have been observed in asthma studies using different methods, including omalizumab (EG2 antibody using non‐stereological image analysis) and mepolizumab (gradient purification).^24^, ^25^ Given the observed variability and treatment group size in this study, approximately 90% reduction in tissue eosinophils would have been needed to reliably be detected. The variability in baseline tissue eosinophils was tenfold higher than assumed for study design power calculations based on BOBCAT study results that used an eosinophil cationic protein antibody, EG2, which stains neutrophils as well.^7^ Notably, neutrophils express eosinophil cationic protein but not EPO.^26^ Anti‐EPO monoclonal antibody MM25‐82.2 was used to detect eosinophils, which is highly specific to eosinophils.^27^ Antibody specificity differences may have contributed to the unexpected higher variability in tissue eosinophils observed in CLAVIER vs BOBCAT.

To ensure high‐quality analyses, quality control on bronchoscopy methods and biopsies was imposed, which resulted in high subepithelial tissue quality. Epithelial tissue quality was lower due to epithelial denudation during the procedure and processing but was consistent with what was expected. Design‐based stereology was used to rigorously quantify eosinophils and demonstrate high measurement precision, suggesting that patient differences contributed to result variability. Reportedly, this is the first application of design‐based stereology in support of an asthma therapeutic clinical trial.

Recent mouse studies raise the possibility of different eosinophil subsets in asthmatic airways distinguished by activation state.^28^ While this pre‐specified stereological enumeration approach did not distinguish activated from non‐activated or regulatory eosinophils, this post hoc non‐stereological pixel‐based quantitation included degranulated signal but was inconclusive due to the high variability. Direct evaluation of tissue eosinophil subpopulations deserves consideration in future studies but will require development and validation of immunohistochemistry markers not currently available.

FEV_1_ changes were clinically meaningful and aligned with FEV_1_ improvements observed after lebrikizumab treatment in phase 3 studies in similar patient populations.^18^ Biomarker subgroups defined by high FeNO and blood eosinophils enriched for FEV_1_ improvement, but high serum periostin did not. Reduction in the biomarker FeNO suggested that lebrikizumab inhibited inflammation in the airways. Trends for decreased *CCL26*, *NOS2* and *SERPINB2* mRNA expression in bronchial tissues were also observed after lebrikizumab treatment, suggesting that IL‐13 blockade reduces chemokines involved in circulation‐to‐airway eosinophil trafficking.^19^

A novel finding in this study is that lebrikizumab reduced the degree of subepithelial fibrosis, a cardinal feature of asthmatic airway remodelling.^29^ Airway subepithelial fibrosis is observed in mouse models after IL‐13 overexpression and in patients with T2 biomarker‐high asthma.^30^, ^31^ Randomized trials previously demonstrated reduction in subepithelial fibrosis with ICS.^32^, ^33^ However, ICS has broad effects, whereas lebrikizumab specifically inhibits IL‐13; so these data mechanistically implicate IL‐13 in subepithelial fibrosis in human asthma. Recently, a bronchoscopic study performed using tralokinumab (another anti‐IL‐13 monoclonal antibody) failed to show this beneficial effect on subepithelial fibrosis.^34^ The reasons for this discrepancy are uncertain but potentially related to differences in measurement methods. Reduction in subepithelial fibrosis was not limited to the T2 biomarker‐high subgroups, raising the possibility that inhibiting lower levels of IL‐13 may be beneficial with respect to remodelling.

The safety profile was consistent with the phase 3 studies in asthma. Two SAEs observed within 3 days of bronchoscopy in patients receiving lebrikizumab (one pneumonia and one leucocytosis) were both considered unrelated to lebrikizumab and due to the bronchoscopy procedure.

This study was limited by incomplete recruitment that reduced statistical power, biomarker subgroup sizes and a treatment arm imbalance by patient race resulting in substantially more Black/African American patients in the lebrikizumab arm than in the placebo arm. Increased risk of eosinophilic inflammation has previously been reported in African American patients on inhaled corticosteroid treatment in a large study of over 1000 patients,^35^ a finding not reflected in our baseline airway subepithelial eosinophils per mm^2^ of basement membrane results (210 cells/mm^2^ for Black/African American patients and 412 cells/mm^2^ for White patients). Other clinical baseline characteristics were similar between Black/African American and White patients (Table S4). This racial imbalance in subepithelial eosinophils is aligned with the overall treatment arm imbalance observed for tissue eosinophils and could potentially be confounding the primary and secondary end‐point results. Randomization for treatment assignment was stratified by serum periostin level and baseline medication but not by blood eosinophil counts nor race. Stratification by blood eosinophil count may have avoided the observed imbalance in tissue eosinophils. The study did not incorporate medication dose counters and adherence to ICS could have impacted the results, though the overall FeNO levels over time in the placebo arm (Figure S9) do not suggest a significant impact of adherence on this relatively short duration study.

In this randomized trial, the effect of lebrikizumab on tissue eosinophils was inconclusive due to baseline imbalances in tissue eosinophils between treatment groups, lack of full recruitment and reduced statistical power, and higher than anticipated variability in tissue eosinophil measurements. Lebrikizumab inhibited the IL‐13 pathway, as demonstrated by changes in key pharmacodynamic biomarkers and was associated with improved lung function and reduced degree of subepithelial fibrosis, a measure of airway remodelling.

## CONFLICT OF INTEREST

All authors report support of the parent study and funding of editorial support from F. Hoffmann‐La Roche. CDA, MGE, RF, MS, MB, KM, JA, DC, JO, FA, KP, MH, KW, FC, WSP and CTJH are employees of Genentech, Inc. JGM was an employee at Genentech, Inc, at the time of the study but is now an employee of 23andMe. PB has received grant funding from Genentech, Inc. GMG has received research funding from Genentech, Inc, paid directly to McMaster University. MF has received research funding from Genentech, Inc, paid directly to UBC. EI has received grants and nonfinancial support from Genentech during the conduct of the study. MK receives research funding for asthma (paid to the University of Arizona) from the National Institutes of Health, American Lung Association, AstraZeneca and Sanofi. MK engages in consulting activities with AstraZeneca and Sanofi and receives royalties from Elsevier. AB has received personal fees and nonfinancial support from Roche outside the submitted work. PW reports fees to his institution for assistance with analysis of samples in this clinical study and personal fees from Amgen, NGM biopharmaceuticals, Theravance, Clarus Ventures, AstraZeneca, 23andMe, Sanofi, Regeneron and GSK outside the submitted work.

## Supporting information

Supplementary Material

## Data Availability

The data sets generated during and/or analysed during the current study are available from the corresponding author on reasonable request. Qualified researchers may request access to individual patient level data through the ClinicalStudyDataRequest platform (www.clinicalstudydatarequest.com). Further details on Roche's criteria for eligible studies are available here (https://clinicalstudydatarequest.com/Study‐Sponsors/Study‐Sponsors‐Roche.aspx). For further details on Roche's Global Policy on the Sharing of Clinical Information and how to request access to related clinical study documents, see here (https://www.roche.com/research_and_development/who_we_are_how_we_work/clinical_trials/our_commitment_to_data_sharing.htm).
