# Supplementary material for: A randomized, placebo‐controlled trial evaluating effects of lebrikizumab on airway eosinophilic inflammation and remodelling in uncontrolled asthma (CLAVIER)
Source: Clin Exp Allergy. 2020 Oct 4;50(12):1342–51. doi: 10.1111/cea.13731 (PMC7756263; doi:10.1111/cea.13731)
Supplement: Supplementary file 1 — Supplementary Material [file CEA-50-1342-s001.docx]

**ONLINE SUPPLEMENT**

**Title:** A randomised, placebo-controlled trial evaluating effects of lebrikizumab on airway eosinophilic inflammation and remodelling in uncontrolled asthma (CLAVIER)

**Authors:** Cary D. Austin, MD, PhD^1^, Melissa Gonzalez Edick, BS^1^, Ronald E. Ferrando, MA^1#^, Margaret Solon, BA^1^, Miriam Baca, BS^1^, Kathryn Mesh, PhD^1^, Peter Bradding, PhD^2^, Gail M. Gauvreau, PhD^3^, Kaharu Sumino, MD^4^, J. Mark FitzGerald, MD^5^, Elliot Israel, MD^6^, Lief Bjermer, MD, PhD^7^, Arnaud Bourdin, MD^8^, Joseph R. Arron, MD, PhD^1^, David F. Choy, BS^1^, Julie K Olsson, MD^1^, Francis Abreu, PhD^1^, Monet Howard, MS^1^, Kit Wong, PhD^1^, Fang Cai, PhD^1^, Kun Peng, PhD^1^, Wendy S. Putnam, PhD^1^, Cécile T. J. Holweg, PhD^1^, John G. Matthews, PhD^1*^, Monica Kraft, MD^9^, Prescott G. Woodruff, MD^10^, *on behalf of the CLAVIER Investigators*

**Author affiliations:** ^1^Genentech, Inc., South San Francisco, CA; ^2^University of Leicester and Glenfield Hospital, Leicester, UK; ^3^McMaster University, Hamilton, Ontario; ^4^Washington University School of Medicine, St Louis, MO; ^5^University of British Columbia, Vancouver, British Columbia; ^6^Brigham and Women’s Hospital, Boston, MA; ^7^Skåne University Hospital, Lund, Sweden; ^8^CHU Montpellier, Montpellier, France; ^9^University of Arizona College of Medicine, Tucson, AZ; ^10^University of California, San Francisco Medical Center, San Francisco, CA. ^#^Now at Stemcentrx/AbbVie, Inc., South San Francisco. *Now at 23andMe, Mountain View, CA

**Corresponding author information:**

Cary Austin, MD, PhD, FCAP

Senior Pathologist, Department of Pathology, gRED

Genentech, Inc., a member of the Roche Group, USA

Austin.cary@gene.com

Phone: 650-225-7357

Fax: 650-467-2625

**METHODS**

**Study design**

Following written informed consent, patients were screened for asthma controller therapy adherence, ability to use the equipment necessary for study clinic visits, and degree of asthma control with their standard-of-care asthma medications. Bronchoscopy was performed at visit 4a to collect baseline samples from patients whose symptoms had remained uncontrolled during screening.

Patients were randomised 1:1 to receive lebrikizumab or placebo through an interactive voice/web-based response system after confirming eligibility criteria and absence of significant bronchoscopy-related adverse events (AEs; e.g., exacerbation requiring corticosteroids and/or hospitalisation). Randomisation was stratified by baseline serum periostin level (<50 or ≥50 ng/mL), baseline asthma medications (total daily dose ≥1000 μg fluticasone propionate dry powder inhaler (DPI) or equivalent plus long-acting beta agonists [LABA; yes, no], and nasosorption/sputum induction substudy participation [yes, no]). A permuted block randomisation method was used to obtain an approximate 1:1 ratio between treatment arms and within each stratum. Patients received treatment on days 1 and 8 and weeks 4 and 8 during the 12-week placebo-controlled period.

**Patients**

Key exclusion criteria were history of a severe allergic reaction or anaphylactic reaction to a biologic agent or known hypersensitivity to any component of the lebrikizumab injection, maintenance oral corticosteroid therapy within 3 months prior to visit 1 or treatment with systemic or intra-articular corticosteroids within 4 weeks prior to visit 1, current infection, tobacco smoking (>10 pack-years), and history of bronchial thermoplasty.

**Outcomes**

Secondary stereologically assessed efficacy endpoints included the following placebo-corrected adjusted mean changes from baseline to week 12: absolute change in number of airway subepithelial eosinophils per mm^2^ of basement membrane, relative and absolute changes in the number of airway subepithelial eosinophils per volume of lamina propria plus submucosa (hereafter referred to as lamina propria), and relative and absolute changes in the number of airway epithelial eosinophils per mm^2^ of basement membrane and μL of epithelium. Assessed IL-13– and asthma-related pharmacodynamic biomarkers included FeNO level; blood eosinophil count; and serum total immunoglobulin E, chemokine (C-C motif) ligand 13, and periostin levels, as well as the mRNA expression of *CCL26*, *CLCA1*, *IL13*, *NOS2*, *POSTN*, and *SERPINB2* in airway epithelial brushings. Change in prebronchodilator FEV_1_ was also reported. The pharmacokinetic outcome measure was post-dose serum lebrikizumab concentration at week 12. Incidence of antitherapeutic antibodies (ATAs) against lebrikizumab was monitored throughout the study up to week 20.

Exploratory remodelling-related stereologically assessed endpoints included relative and absolute changes in the thickness of subepithelial collagen, volume of stored mucin per mm^2^ of basal lamina, and number of goblet cells per mm^2^ of basal lamina. An exploratory non-stereological endpoint included relative and absolute changes in mean optical density of anti–eosinophil peroxidase (EPO) immunohistochemistry signal per section image pixel of lamina propria and epithelium.

**Procedures**

All sites were trained in the same methodology and procedures. The order of processing of the biopsies was predefined to allow for the collection of biopsies for histology (≤6 biopsies) as well as for gene expression assessment (≤2 biopsies) for a maximum of 8 biopsies per patient. Quality criteria were predefined for all sample types and assessments, and quality review of the samples was performed monthly by the Biospecimen Quality Assurance Group, consisting of key representatives from the University of California, San Francisco Airway Clinical Research Center and Genentech/Roche. Real-time feedback was provided to sites not meeting the criteria to ensure that training was repeated for new site staff or sites not meeting quality metrics.

Bronchoscopy, collection of epithelial brushings and ≤8 subsegmental bronchial biopsies (approximately 2 mm in diameter), and sample processing were performed per the study procedure manual. Patients were not allowed to eat or drink for ≥8 hours and could not have taken aspirin, anticoagulant, or antiplatelet medication within 7 days before the procedure. If the patient experienced a significant bronchoscopy-related adverse event at the first bronchoscopy, the patient was not entered into the study.

Immunohistochemistry was optimised using a robust anti–eosinophil peroxidase (EPO) antibody (monoclonal antibody [mAb] MM25-82.2 I) [1]. This antibody was used because it did not show cross-reactivity with neutrophils, whereas another commonly used antibody for detecting eosinophils in human tissue, anti–eosinophil cationic protein antibody mAb EG2, did show neutrophil cross-reactivity (**Figure E1**). Tissue eosinophils were quantified using design-based stereology, an analytic approach endorsed as the gold standard for lung quantitative microscopy by the American Thoracic Society and the European Respiratory Society.[2]

Lebrikizumab 125 mg or placebo was administered subcutaneously on days 1 and 8 and weeks 4 and 8 during the 12-week placebo-controlled period. Patients continued to receive stable doses of their standard-of-care therapy, which included inhaled corticosteroid therapy and a second controller medication. The day 8 study drug dose was included to facilitate attainment of steady-state drug levels during the 12-week treatment period. The dosing frequency was supported by the half-life of lebrikizumab of approximately 25 days, as well as safety and efficacy data from previous phase 2 studies [3-5]. Based on previous phase 2 studies, a dose/regimen of 125 mg every 4 weeks was expected to achieve drug concentrations sufficient to inhibit interleukin 13 [4, 5].

Assessments during the study included demographics and clinical characteristics, pulmonary function and fractional exhaled nitric oxide (FeNO), bronchoscopy samples, adverse events (AEs), pharmacokinetics, pharmacodynamic biomarkers, and antitherapeutic antibodies (**Table E1**). Safety assessments included monitoring the frequency and severity of treatment-emergent AEs, incidence of antibodies against lebrikizumab throughout the study, electrocardiograms, and vital signs. Treatment-emergent AEs were defined as any new AE reported or any worsening of an existing condition on or after the first dose of study drug. Serum lebrikizumab concentration was analysed at week 12.

**Blinding to pharmacodynamic biomarkers**

Patients and all study-site personnel were blinded to periostin levels, blood eosinophil counts, and other pharmacodynamic biomarker levels until study completion, except as described. Sponsor study team members who had direct contact with study sites remained blinded to individual periostin and FeNO levels, but team members who did not interact with study sites could review individual pre-treatment serum periostin and FeNO levels for assessment of baseline study information and ongoing review of data quality. The sponsor remained blinded to individual posttreatment periostin and other pharmacodynamic biomarker levels until completion of the placebo-controlled period.

Blood eosinophil counts during screening were reviewed by the sites and the sponsor as part of routine laboratory data review for eligibility. Patients, all study-site personnel, and the sponsor (with the exception of the internal monitoring committee) were blinded to the eosinophil counts after randomisation except for clinically significant elevations in eosinophil counts, which were reported to sites and could be reviewed by the sponsor as part of routine safety monitoring.

**Asthma exacerbations**

An asthma exacerbation was defined as new or increased asthma symptoms (including wheeze, cough, dyspnoea, chest tightness, and/or night-time awakening due to these symptoms) that led to treatment with systemic corticosteroids or to hospitalisation. Treatment with corticosteroids was defined as treatment with oral, intravenous (IV), or intramuscular (IM) corticosteroids for ≥3 days or an emergency department visit with ≥1 dose of IV or IM corticosteroids. Patients who experienced an exacerbation within 4 weeks prior to visit 8 did not undergo bronchoscopy at the week 12 visit.

**Fractional exhaled nitric oxide**

Measurement of FeNO was performed using a handheld portable NIOX MINO device (Aerocrine, Solna, Sweden) in accordance with guidelines published by the American Thoracic Society and described in the Pulmonary Function Testing Manual. FeNO measurement was performed prior to spirometry testing**.** The FeNO device did not display the test result, and the sites remained blinded to FeNO.

**Spirometry**

Spirometry, including the procedure for bronchodilator testing, was conducted per the study Pulmonary Function Testing Manual. Measurement was performed on a computerised spirometry system, Vitalograph Spirotrac, with 6800 Spirometer (Vitalograph, Ennis, Ireland) configured to the requirements of the study and in accordance with guidelines published by the American Thoracic Society/European Respiratory Society.[6] Spirometry measures included forced expiratory volume in 1 second (FEV_1_), forced vital capacity (FVC), and peak expiratory flow, and flow-volume and volume-time curves were generated. The percentage of predicted FEV_1_ and predicted FVC were derived from volume measurements using equations derived from the third National Health and Nutrition Examination Survey [7]. A repeat visit 2 spirometry session was performed if the visit 2 spirometry was rejected by the over-reader, who determined the acceptability of the data, and visit 2 was the only session that could be repeated. Pre- and postbronchodilator spirometry were performed according to the schedule of assessments, but spirometry could be omitted if a patient was experiencing an acute asthma exacerbation at the time of the scheduled study visit.

**Bronchoscopy, including brushings and biopsies**

Bronchoscopy, including brushings and biopsies, was performed per the study procedure manual. Patients were nil per os for ≥8 hours before the procedure and could not have taken aspirin, anticoagulant, or antiplatelet medication within 7 days before the procedure. However, the use of nonsteroidal anti-inflammatory drugs was not restricted. If the patient experienced a significant bronchoscopy-related AE at the first bronchoscopy (e.g., exacerbations requiring steroids and/or hospitalisation), the patient was excluded from the study.

Flexible fibreoptic bronchoscopy was performed at visits 4a and 8, including epithelial brushings and collection of endobronchial biopsy samples using either a reusable Pentax cat# KH-2411S or a disposable Olympus cat# FB-231D.A forceps. If the patient had received systemic corticosteroids in the previous 4 weeks or was experiencing an acute asthma exacerbation event at the time of the scheduled visit 8, bronchoscopy was not performed. The biopsy procedure called for 8 biopsies, 6 of which were designated for histological processing and 2 designated for RNA extraction and analysis.

Biopsy processing for histology was standardised across all sites, with strict control of extent of formalin fixation (4 hours at 4°C). Routine methods were used for biopsy processing and paraffin embedding. All biopsies from each bronchoscopy were arrayed by the sponsor within a single paraffin block using a stereological “isector” to enable isotropic, uniform random sections as described and analysed for the following: number of airway subepithelial and epithelial eosinophils (cells/mm^2^ of basement membrane surface area and cells/μL of lamina propria and epithelial tissue) and gene expression in airway epithelial brushing and biopsy samples.[8]

**Special stains and immunohistochemistry**

For biopsy quality and airway epithelial mucin evaluations, histological sections were stained with periodic acid-Schiff-alcian blue. For measurements of the thickness of airway subepithelial collagen, histological sections were stained with Gomori trichrome. Immunohistochemistry was performed on 3-μm semiserial sections of biopsy arrays adequate for sampling of lamina propria, epithelium, or both using EPO-specific monoclonal antibody MM25-82.2 with no antigen retrieval using a BOND III autostainer and a Bond Polymer Refine Detection kit (Leica Biosystems, Wetzlar, Germany) according to the manufacturer’s instructions [9]. The anti–eosinophil cationic protein monoclonal antibody EG2 was not used, as it showed cross-reactivity with neutrophils (**Figure E1**).

**Stereological and non-stereological analyses**

Slides were digitally scanned at 20× using a NanozoomerXR scanner (Hamamatsu Photonics, Japan). Tissue eosinophils, airway epithelial mucin volume, and thickness of subepithelial collagen were quantitated using design-based stereology, an analytic approach endorsed as the gold standard for lung quantitative microscopy by the American Thoracic Society and the European Respiratory Society [2]. Images were stereologically analysed within intact portions of lamina propria and/or epithelium by adopting previously described approaches to NewCAST software, version 5 (VisioPharm, Denmark) [8, 10, 11]. Briefly, for eosinophil enumeration, immunoreactive lamina propria and epithelial eosinophils were separately enumerated in randomly generated fields of view, together with enumeration of grid points overlying lamina propria and epithelium and grid lines intersecting lamina propria. Eosinophils were counted using the physical disector method, counting only those eosinophils appearing on one or the other semiserial sections, but not on both (i.e., bidirectional reference and look-up sections). Estimates of the number of eosinophils per volume of lamina propria and epithelium (Nv) were each calculated using the formula, $Nv= \frac{\sum Q}{n \times BA \left( \frac{a}{P} \right)\sum P}$, where Q is the eosinophil count, n is section distance number, BA is block advance (3 μm), a/P is area per grid point, and P is the total number of grid points counted. Lamina propria and epithelial eosinophils per mm^2^ of basement membrane were then calculated by multiplying estimates of the lamina propria surface area per volume of epithelium or lamina propria. These surface area per volume estimates were calculated using the formula, $Sv=\frac{2\sum I}{\frac{l}{p}\sum P}$, where I is the line intercept with lamina propria count, l/p is the length of the line per grid point, and P is grid point count. As an exploratory analysis, MM25.82.2 immunohistochemistry images were non-stereologically analysed in MATLAB (version R2017b by Mathworks, Natick, MA) as 24-bit RGB images; intact subepithelial and epithelium regions were manually annotated in biopsies that passed a QA assessment and mean optical density of DAB staining per pixel determined separately for subepithelial and epithelial regions using a blue-normalised algorithm to identify brown pixels [12].

**Quality control of samples**

Biopsies were pre-screened histologically using periodic acid-Schiff-alcian blue staining for sampling adequacy by specific analysis inclusion criteria. The lamina propria analysis criteria include ≥2 biopsies each with at least one 400× magnification field area of intact lamina propria and, cumulatively, at least ten 400× magnification fields of intact lamina propria in the array. The epithelium analysis criteria included ≥2 biopsies in the array with at least two-thirds of a 400× magnification field width of intact epithelium and, cumulatively, at least six 400x magnification field widths of intact epithelium in the array.

Quality of mRNA of biopsies and brushings for gene expression was determined using a bioanalyser and analyses was prespecified as RNA integrity number of ≥5.0.

**Statistical analyses**

The planned sample size (n=80 with 40 patients per treatment arm) was calculated to provide 74% power to detect a treatment benefit of 50% reduction in the number of airway subepithelial eosinophils per mm^2^ of basement membrane (cells/mm^2^) from baseline to week 12 under the following assumptions: standard deviation of 30 cells/mm^2^ as observed by Jia et al,[13] baseline mean tissue eosinophil count of 55 cells/mm^2^, 2-sided α of 0.05, and 17% loss of data at week 12 (e.g., due to sample quality and/or dropouts). Because approximately 50% of enrolled patients were expected to have a baseline serum periostin level of ≥50 ng/mL, a sample size of 20 patients per treatment arm per periostin subgroup was needed for 72% power to detect a treatment benefit of a 70% reduction from baseline to week 12 in tissue eosinophils based on the variability and count assumptions detailed above.

Three analysis populations were defined for this study. The intent-to-treat population was defined as all patients who were randomised into the study. The safety-evaluable population was defined as all patients who received ≥1 dose of study drug. The primary analysis population was defined as the subset of patients from the intent-to-treat population who received ≥1 dose of study drug and who had evaluable biopsies for both baseline and week 12 visits.

Patients were classified into periostin biomarker subgroups according to their values at visit 1 (day −21). Periostin high was defined as ≥50 ng/mL, and periostin low was defined as <50 ng/mL. For exploratory biomarker analyses, patients were classified into subgroups based on blood eosinophil counts and FeNO values at visit 1. Eosinophil high was defined as ≥300 cells/μL, and eosinophil low was defined as <300 cells/μL. FeNO high was defined as ≥30 ppb, and FeNO low was defined as <30 ppb.

Unadjusted descriptive summaries of primary, secondary, and exploratory endpoints were presented by treatment and time points. Adjusted analyses of absolute and relative changes in tissue eosinophils were conducted using linear regression models with response variables relative or absolute change as appropriate and including the following covariates: treatment arm (lebrikizumab or placebo), history of asthma exacerbations within 12 months prior to study entry (0, ≥1), and baseline asthma medications (inhaled corticosteroid total daily dose ≥1000 μg of fluticasone propionate dry powder inhaler or equivalent plus long-acting beta agonist [yes, no]). Demographic and baseline characteristics, number of exacerbations in the prior year, concomitant medication use, pulmonary function, and asthma control (as measured by the ACQ-5) were summarised by treatment arm and periostin subgroups with descriptive statistics. Lebrikizumab serum concentrations at week 12 were summarised by descriptive statistics. Study drug exposure was summarised by treatment arm, both overall and by periostin subgroups.

**SUPPLEMENTAL FIGURES**

**Figure E1.** Initial immunohistochemistry optimisation experiments revealed that neutrophils present in routinely fixed acute bronchopneumonia lung tissue (A) and an asthmatic endobronchial biopsy fixed identically to CLAVIER biopsies (B and C) express detectable eosinophil cationic protein as detected by mAb EG2 (A and B) but not EPO as detected by mAb 673 (C and not shown). To improve EPO signal detection, we switched to a more sensitive antibody, mAb MM25-82.2, which is robustly detected in eosinophils but not neutrophils in the same acute bronchopneumonia (D) and asthmatic endobronchial biopsies (E). EPO, eosinophil peroxidase; mAb, monoclonal antibody.

**
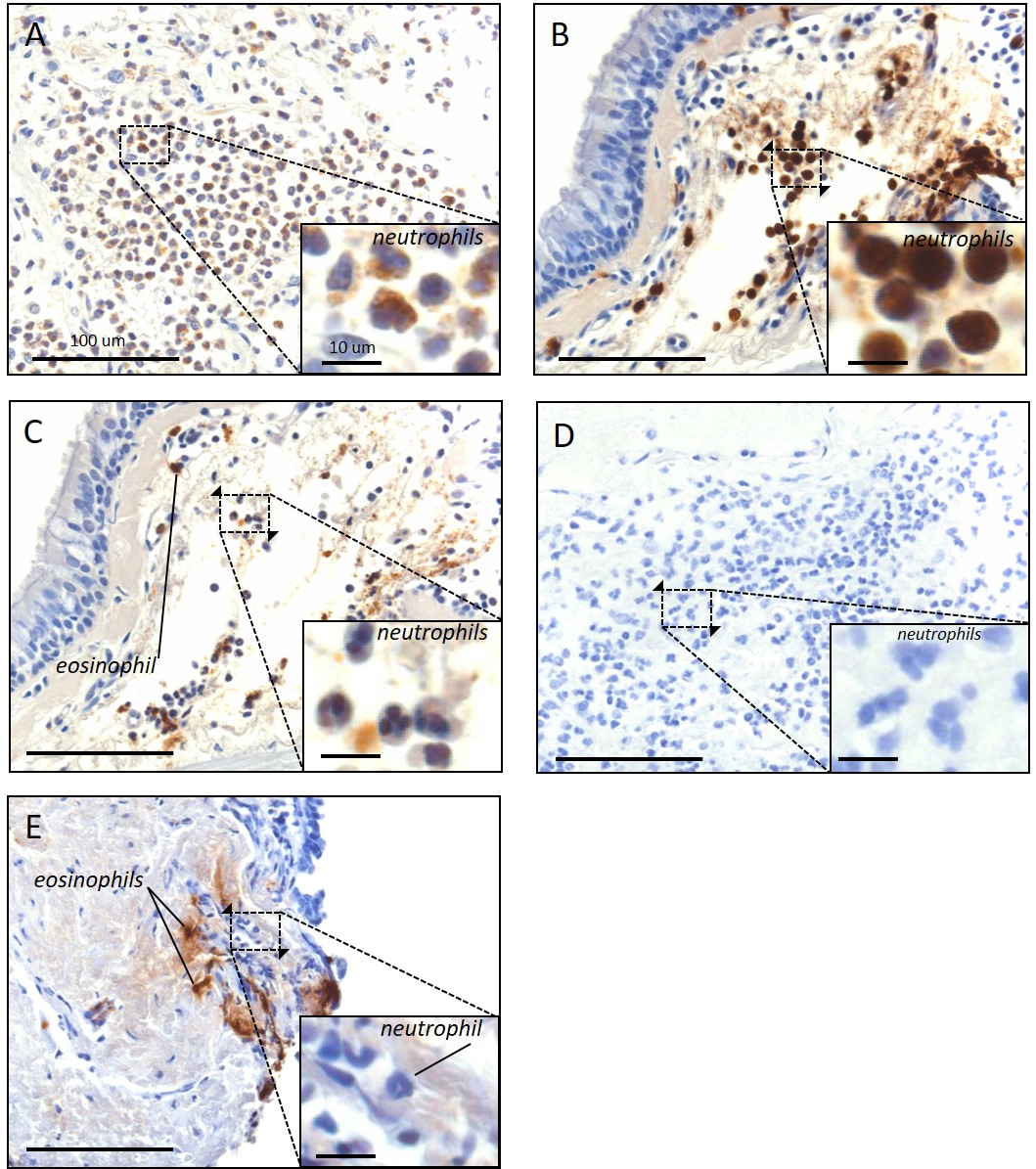
**

**Figure E2.** Unadjusted data corresponding to the primary endpoint. Relative (%) changes from baseline in number of airway subepithelial eosinophils per mm^2^ of basement membrane at week 12 based on biomarker subgroup. Relative change was defined as the absolute change from baseline to week 12 divided by the value at baseline. FeNO, fractional exhaled nitric oxide; L125, lebrikizumab 125 mg; PLBO, placebo.


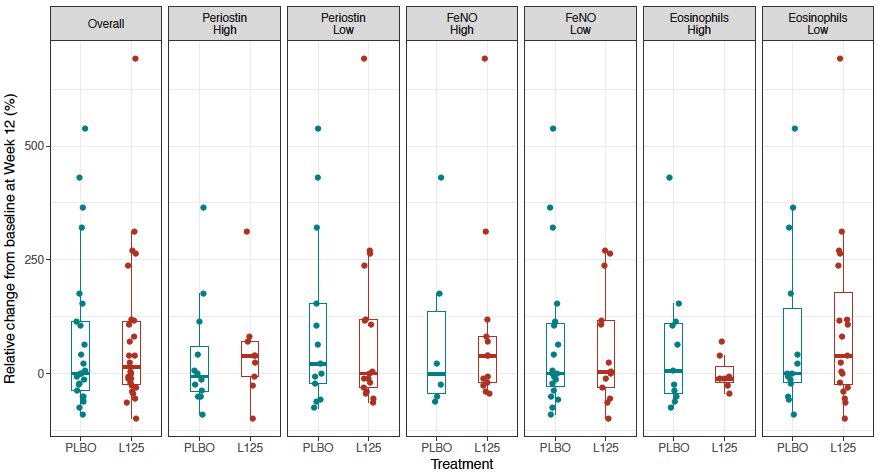


**Figure E3.** Secondary efficacy endpoint. Mean adjusted absolute changes from baseline in number of airway subepithelial eosinophils per mm^2^ of basement membrane at week 12 in the primary analysis population. Estimates were based on a linear model that used relative change from baseline in airway subepithelial eosinophils as the response variable and included terms for treatment, number of asthma exacerbations within 12 months of study entry, and baseline asthma medications. Placebo-corrected adjusted mean change is the difference in adjusted mean changes between the lebrikizumab and placebo groups. FeNO, fractional exhaled nitric oxide.


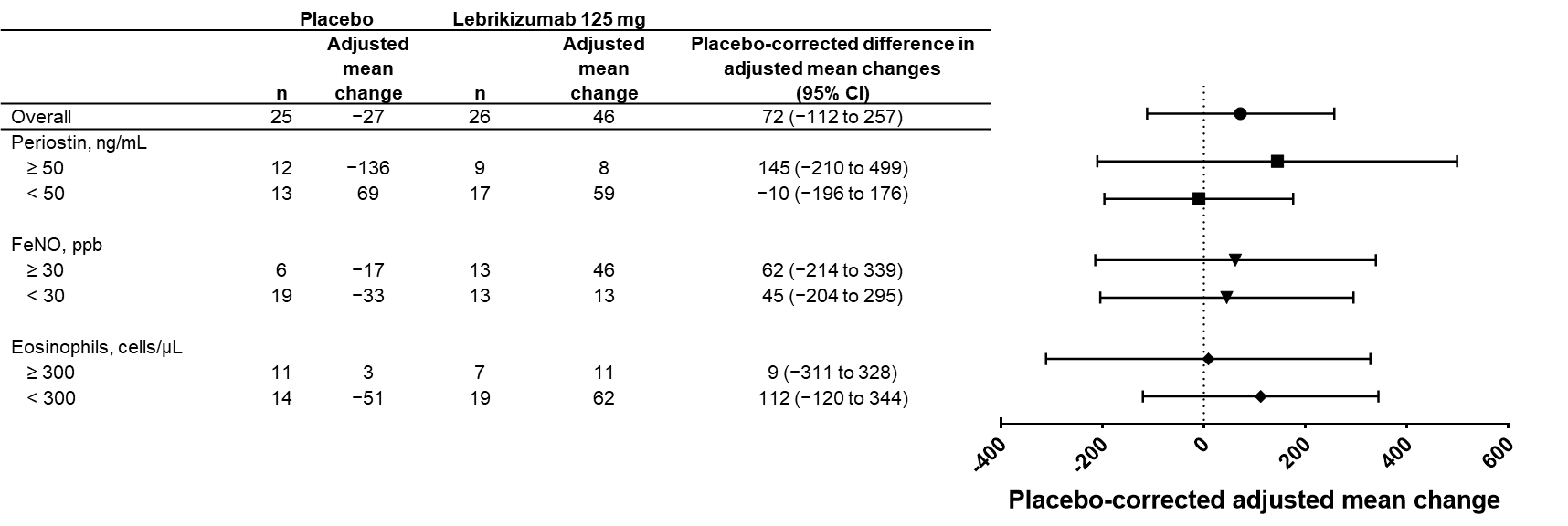


**Figure E4.** Secondary efficacy endpoint. Relative (%) and absolute adjusted changes in number of airway subepithelial eosinophils per volume of lamina propria (cells/μL). Relative change was defined as the absolute change from baseline to week 12 divided by the value at baseline. Placebo-corrected adjusted mean change is the difference in adjusted mean changes between the lebrikizumab and placebo groups. FeNO, fractional exhaled nitric oxide.

1. Absolute change, cells/μL

**
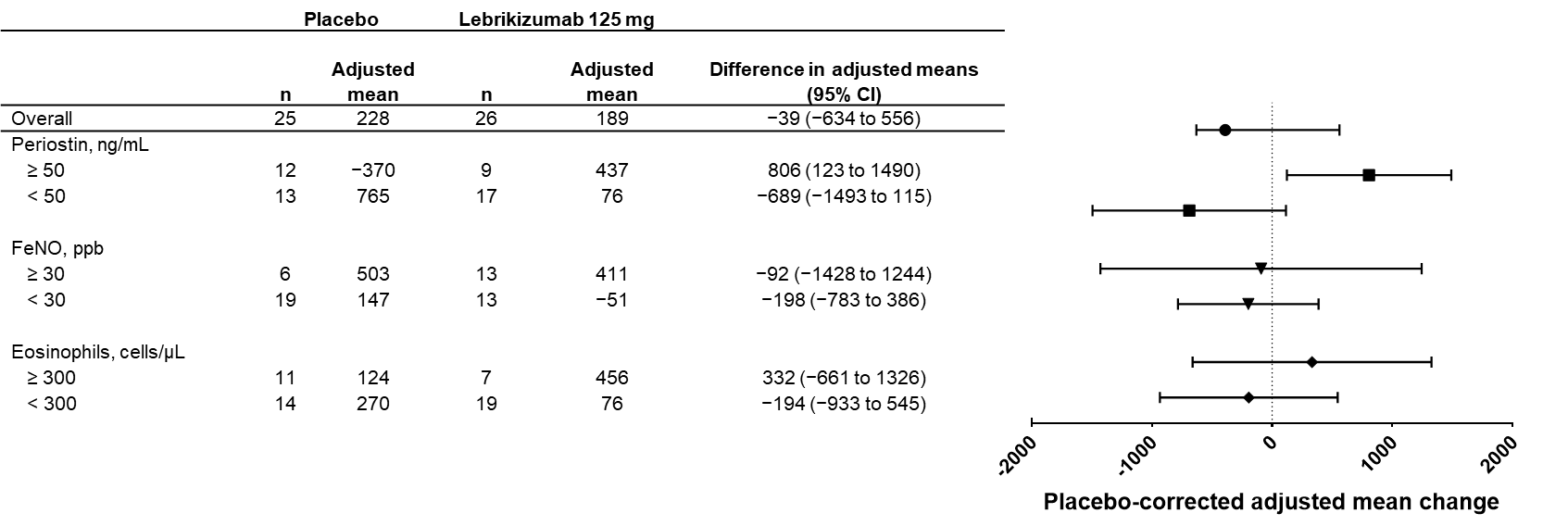
**

1. Relative change, %

**
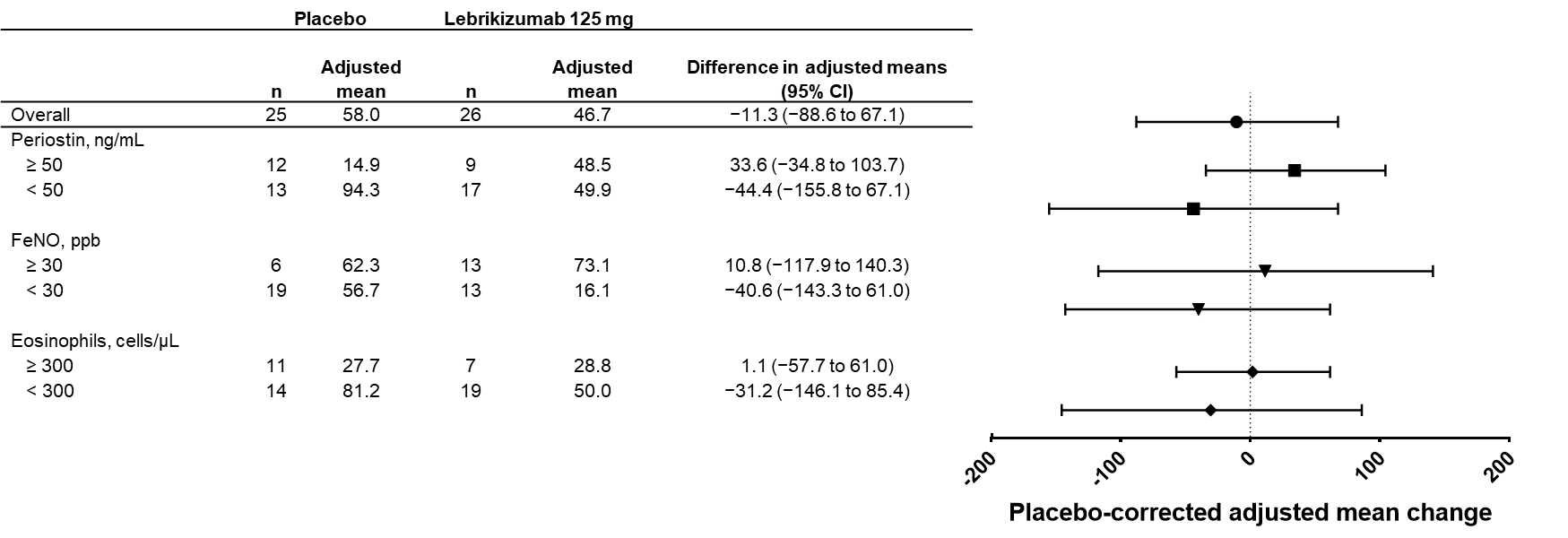
**

**Figure E5.** Secondary efficacy endpoint. Relative (%) and absolute adjusted changes in FeNO and FEV_1_ based on biomarker subgroup. Relative change was defined as the absolute change from baseline to week 12 divided by the value at baseline. Placebo-corrected adjusted mean change is the difference in adjusted mean changes between the lebrikizumab and placebo groups. FeNO, fractional exhaled nitric oxide; FEV_1_, forced expiratory volume in 1 second.

1. Absolute change in FEV_1_, L


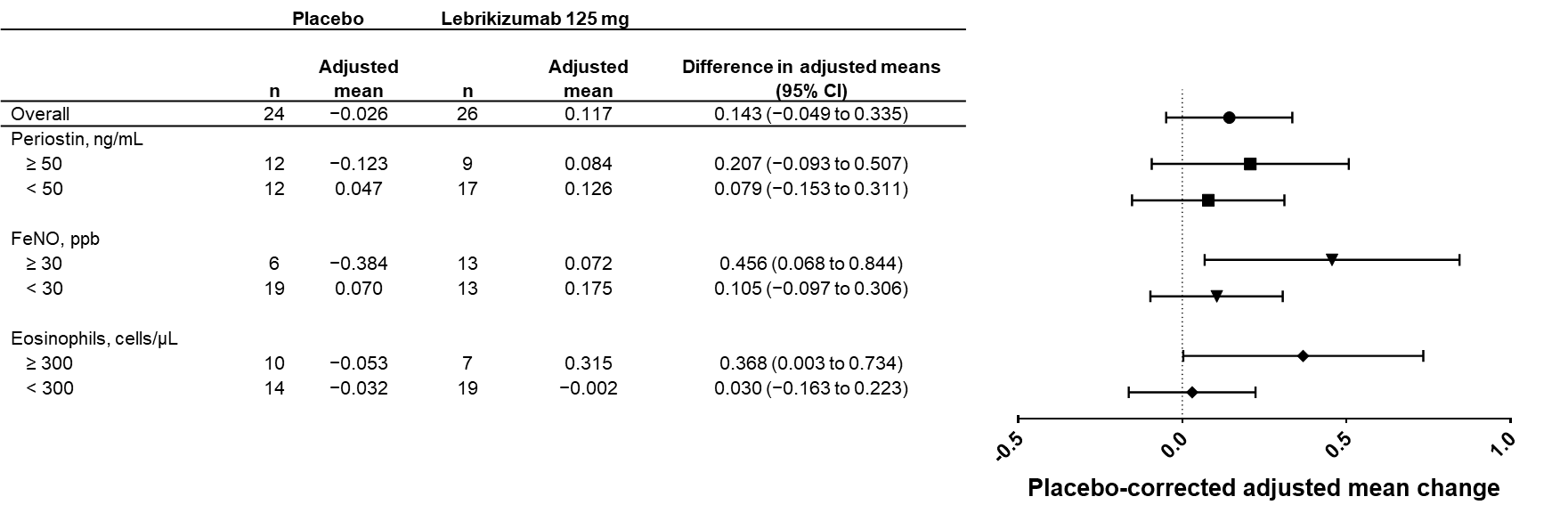


1. Relative change in FEV_1_, %


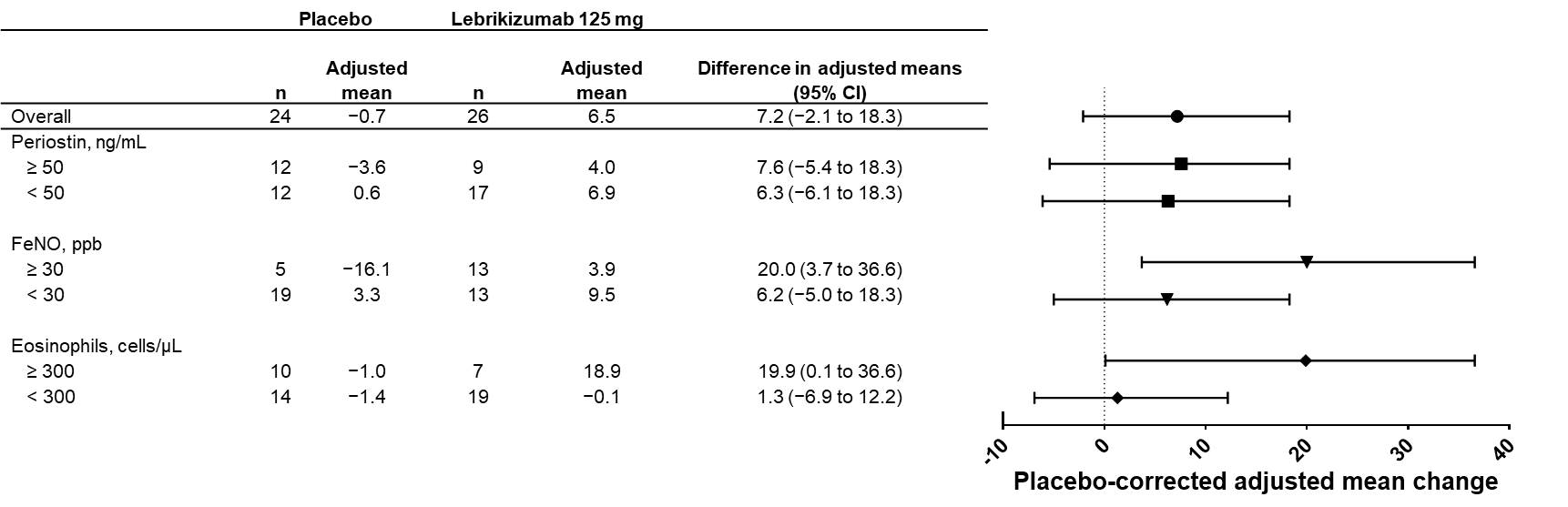


1. Absolute change in FeNO, ppb


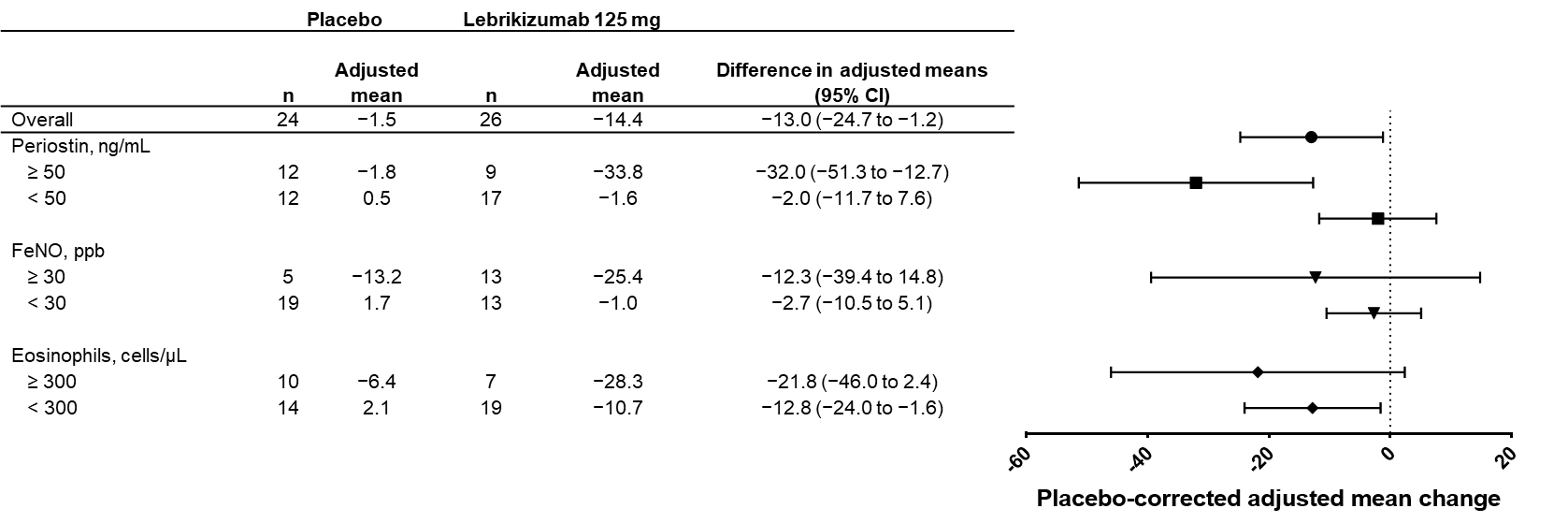


1. Relative change in FeNO, %


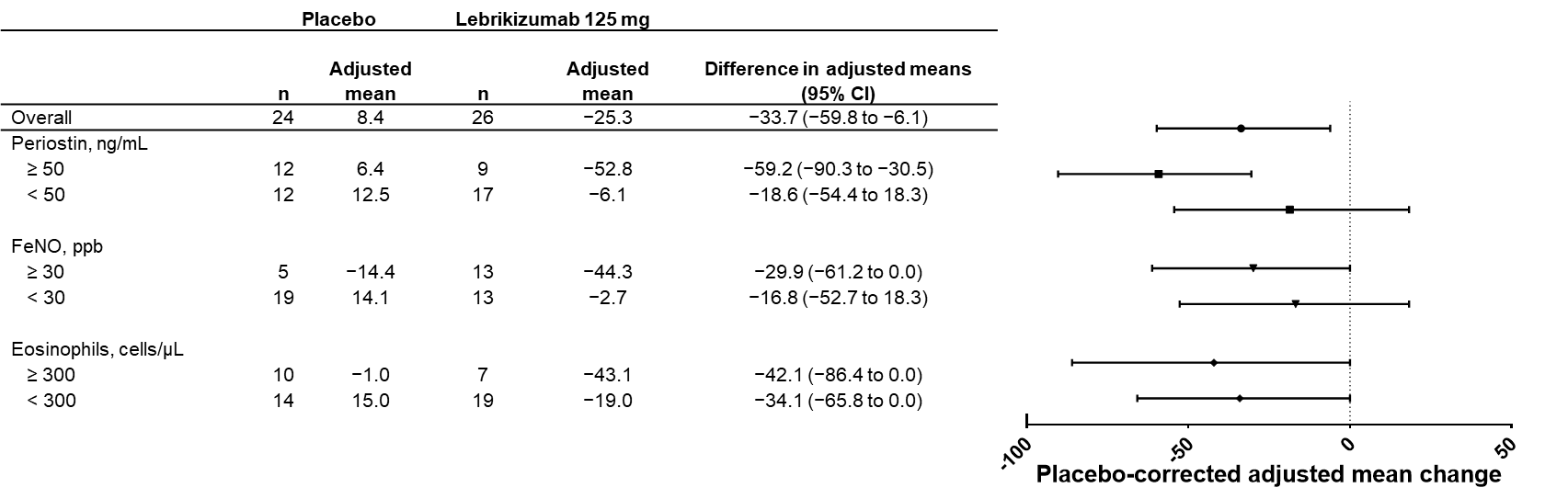


**Figure E6.** Exploratory histological endpoints. Relative (%) and absolute changes in mean OD per EPO immunohistochemistry image pixel based on biomarker subgroup. Relative change was defined as the absolute change from baseline to week 12 divided by the value at baseline. EPO, eosinophil peroxidase; FeNO, fractional exhaled nitric oxide; L125, lebrikizumab 125 mg; OD, optical density; PLBO, placebo.

1. Relative change in mean OD per pixel of subepithelium, %


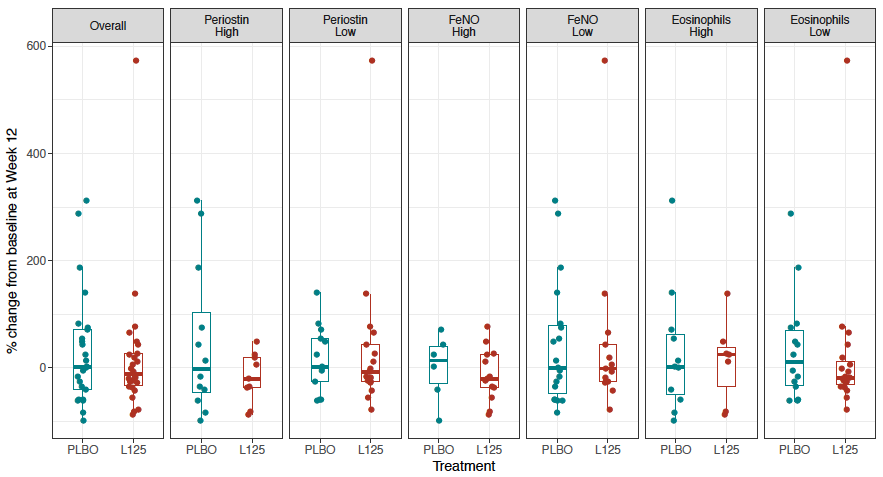


1. Absolute change in mean OD per pixel of subepithelium


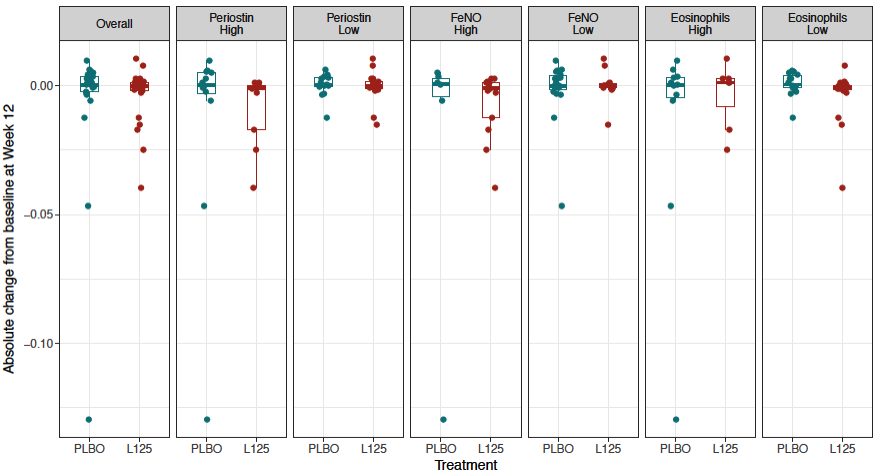


1. Relative change in mean OD per pixel of epithelium, %. One outlier data point (lebrikizumab treated, periostin low, FeNO low, eosinophils low) lies beyond the axis scale to facilitate data visualisation.


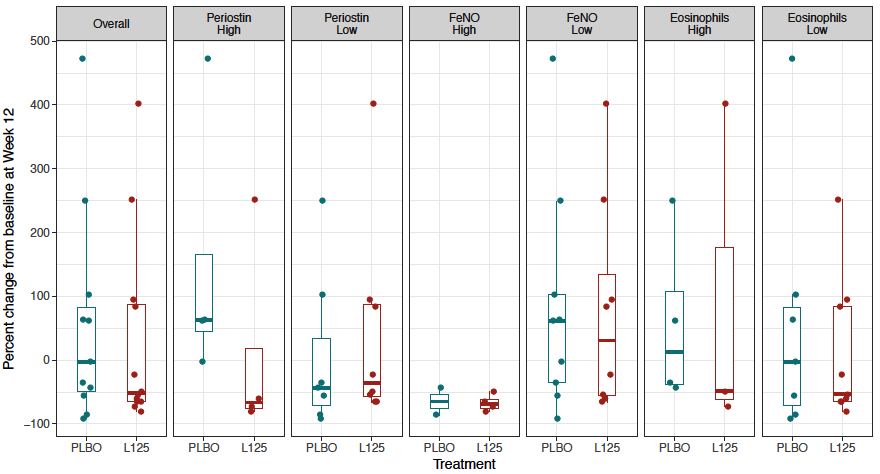


1. Absolute change in mean OD per pixel of epithelium
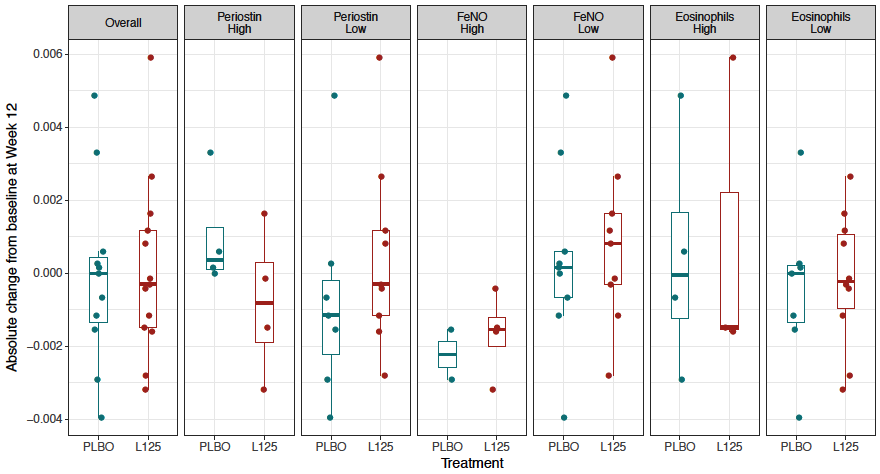


**Figure E7.** Exploratory histological endpoints. Relative (%) and absolute changes in thickness of subepithelial collagen based on biomarker subgroup. Relative change was defined as the absolute change from baseline to week 12 divided by the value at baseline. FeNO, fractional exhaled nitric oxide; L125, lebrikizumab 125 mg; PLBO, placebo.

1. Relative change in mean thickness of subepithelial collagen, %


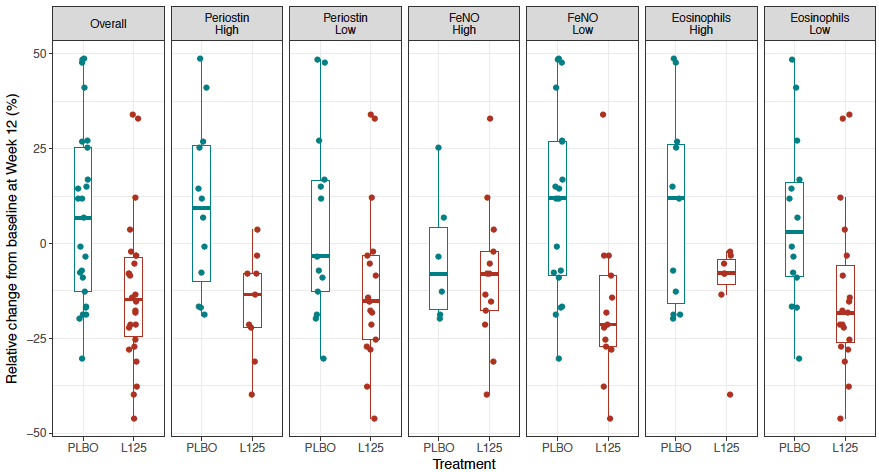


1. Absolute change in mean thickness of subepithelial collagen, μm


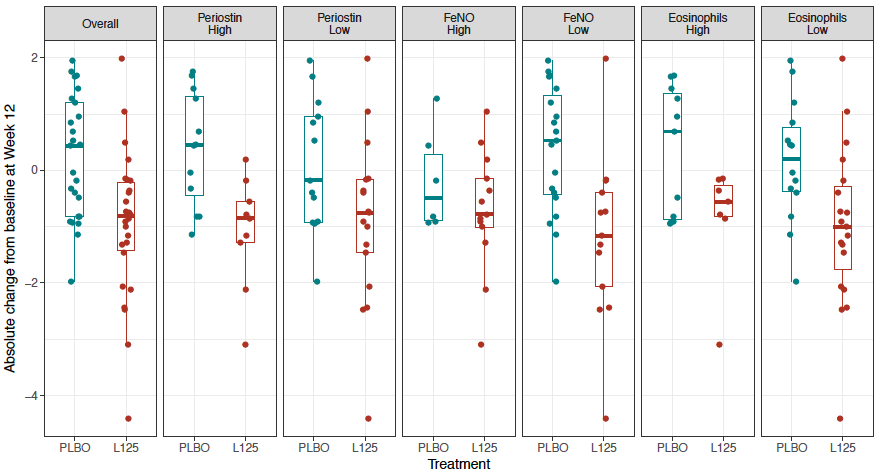


**Figure E8.** Exploratory histological endpoints. Relative (%) and absolute changes in volume of epithelial stored mucin and number of goblet cells per mm^2^ of basement membrane based on biomarker subgroup. Relative change was defined as the absolute change from baseline to week 12 divided by the value at baseline. FeNO, fractional exhaled nitric oxide; L125, lebrikizumab 125 mg; PLBO, placebo.

1. Relative change in volume of epithelial mucin per mm^2^ of basement membrane, %


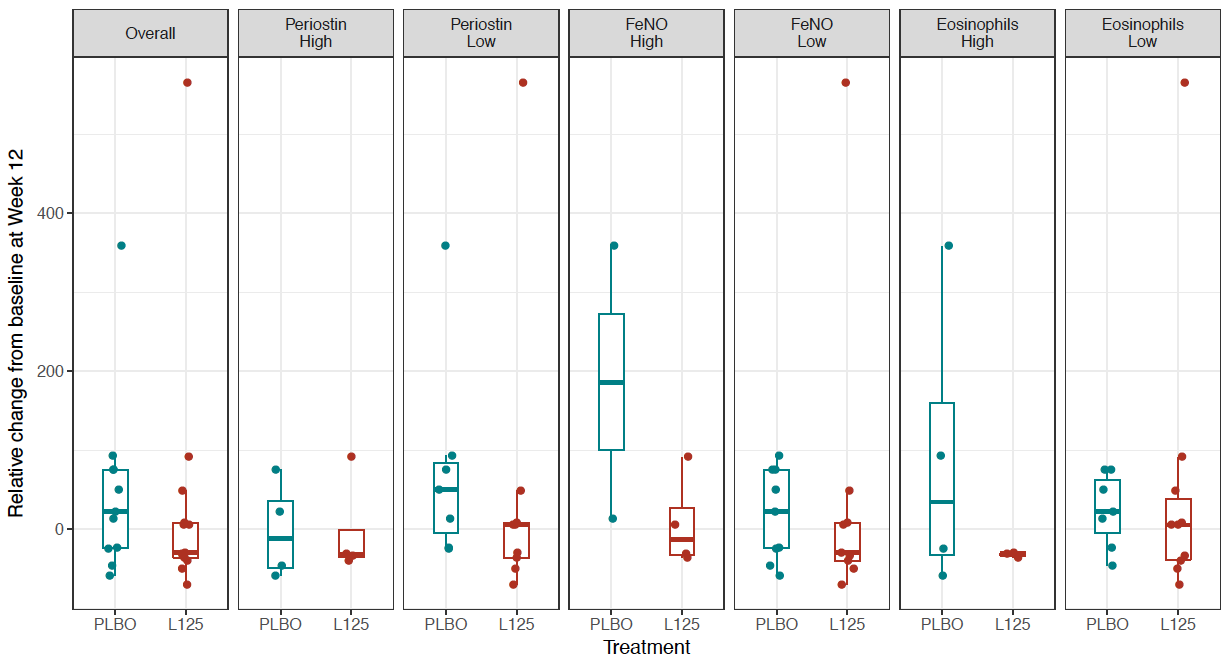


1. Absolute change in volume of epithelial mucin per mm^2^ of basement membrane, %
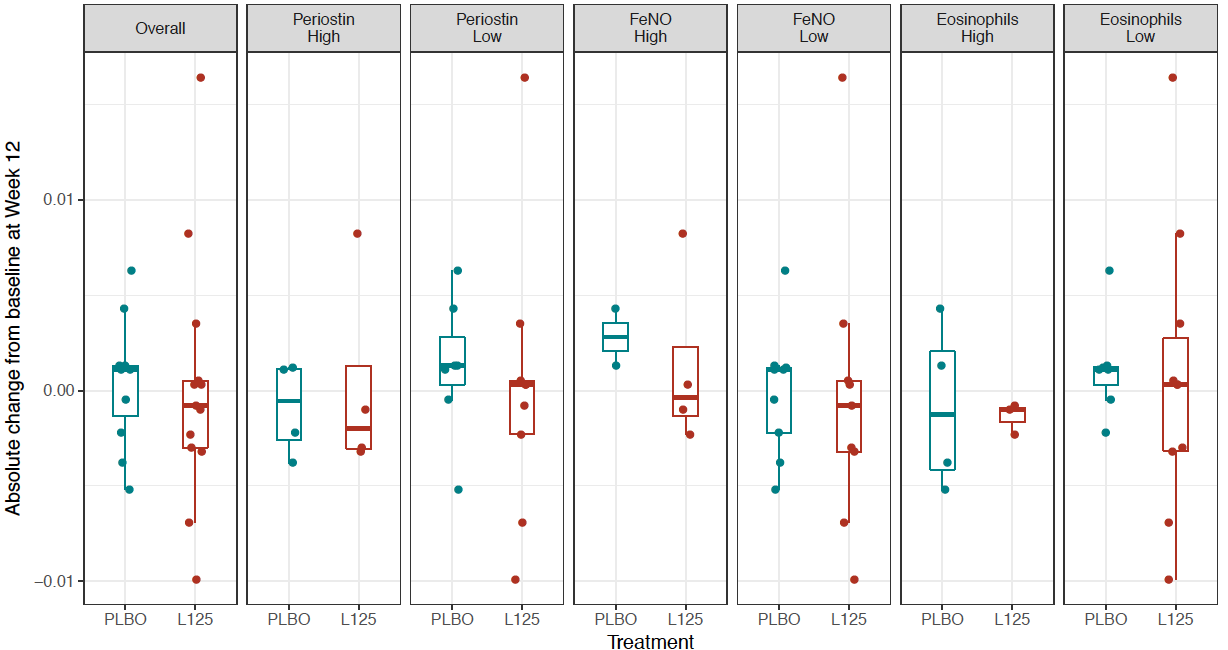

2. Relative change in number of goblet cells per mm^2^ of basement membrane, %


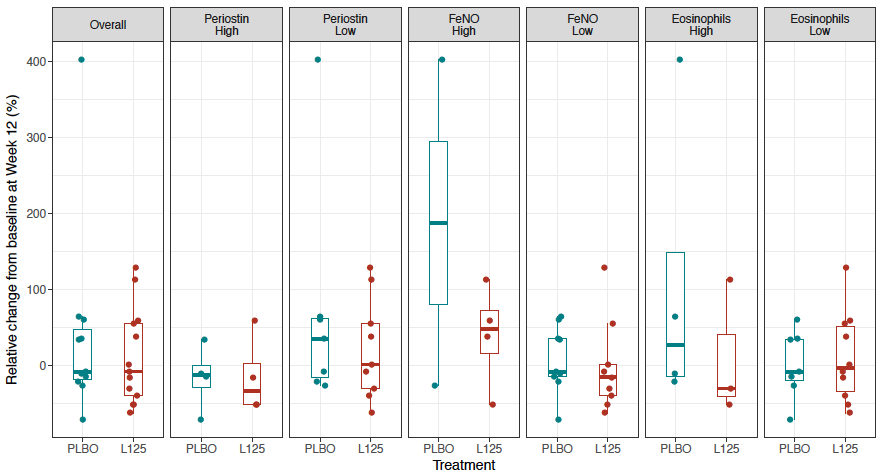


1. Absolute change in number of goblet cells per mm^2^ of basement membrane, mm^−2^

**Figure E9.** Median change from baseline in key blood IL-13–related biomarkers in the safety-evaluable population. CCL, chemokine ligand; Ig, immunoglobulin; IL, interleukin; FeNO, Fractional exhaled Nitric Oxide

1. Percent change in serum CCL-13


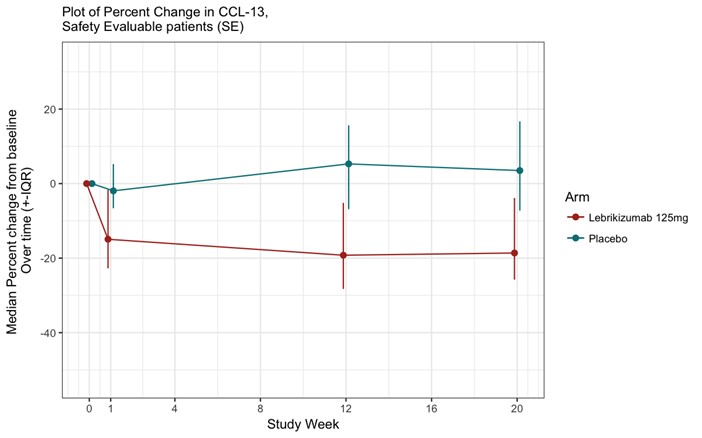


1. Percent change in serum periostin


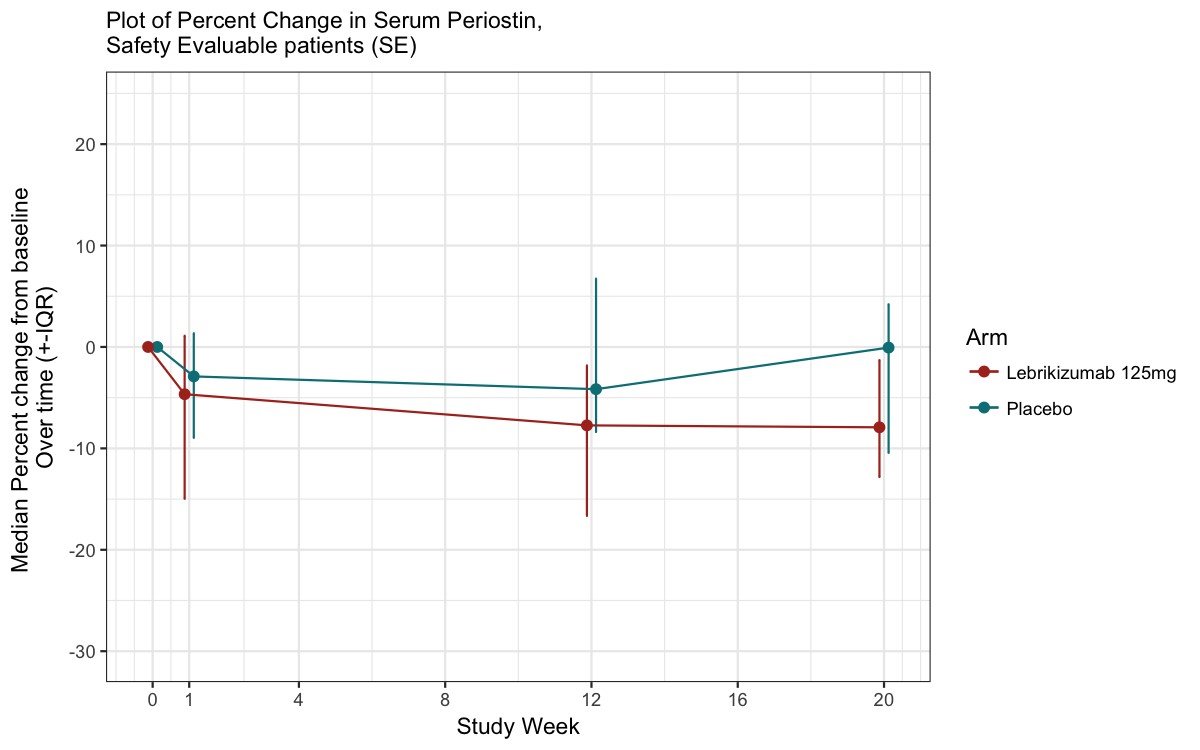


1. Percent change in serum total IgE


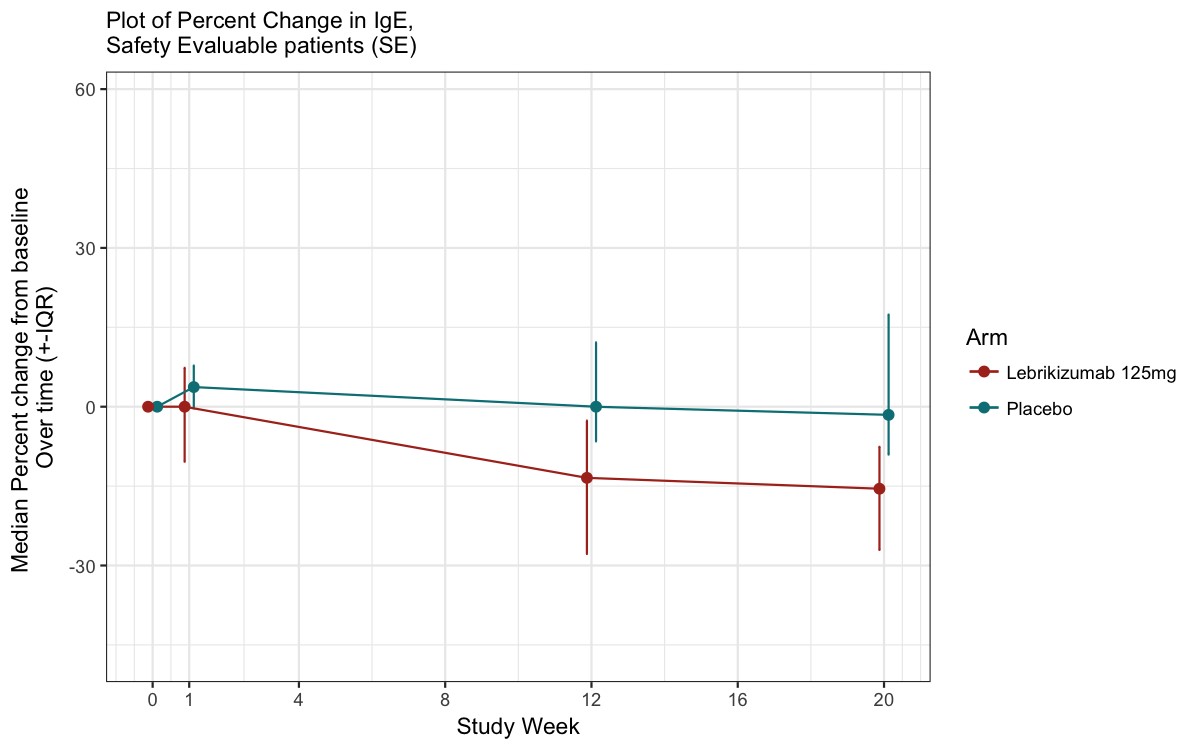


1. Absolute change in blood eosinophils

**
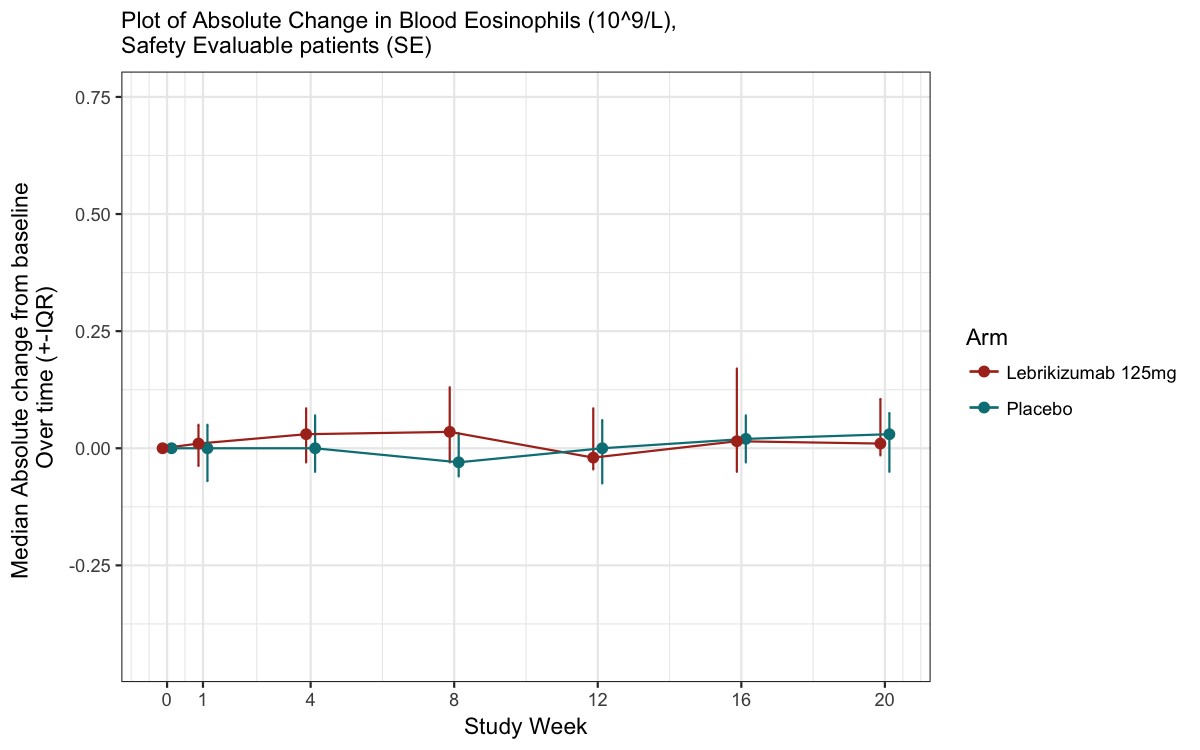
**

1. Absolute change in FeNO

**
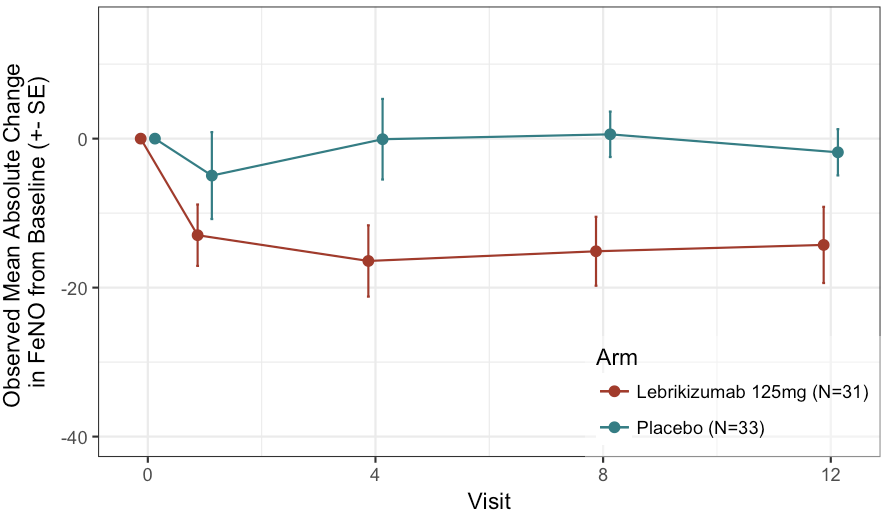
**

**Figure E10.** Fold change at week 12 in respiratory epithelium mRNA expression (RPKM) in the primary analysis population. Fold change from baseline was calculated as log_2_(value at week 12 + 0.01) – log_2_(value at baseline + 0.01). Log_2_ corresponds to logarithm in base 2. A small quantity (0.01) was added to each value to deal with log_2_(0) values. FeNO, fractional exhaled nitric oxide; L125, lebrikizumab 125 mg; PLBO, placebo.

1. *CCL26*

1. *NOS2*

1. *SERPINB2*

1. *IL13*

1. *CLCA1*

1. *POSTN*

**SUPPLEMENTAL TABLES**

**Table E1.** Schedule of assessments

| **Procedure** | **Screening period** | | | | **Placebo-controlled period** | | | | | **Follow-up period** | | **Dosing termination** | **Early termination** | **Unscheduled** |
| --- | --- | --- | --- | --- | --- | --- | --- | --- | --- | --- | --- | --- | --- | --- |
| Visit | 1 | 2 | 3 | 4a* | 4b^†^ | 5 | 6 | 7 | 8 | 9 | 10 |  |  |  |
| Week | – | | | | – | 1 | 4 | 8 | 12 | 16 | 20 |  |  |  |
| Day | −21 | −14 | −7 | – | 1 | 8 | 29 | 57 | 85 | 113 | 141 |  |  |  |
| Window, days | – | +3 | +3 | +3 | – | ±3 | ±3 | ±3 | ±4 | ±3 | ±3 |  |  |  |
| Informed consent | X |  |  |  |  |  |  |  |  |  |  |  |  |  |
| Demographics | X |  |  |  |  |  |  |  |  |  |  |  |  |  |
| Medical history^‡^ | X |  |  |  |  |  |  |  |  |  |  |  |  |  |
| Physical examination | X |  |  |  |  |  |  |  |  |  |  |  |  |  |
| Weight | X |  |  |  |  |  |  |  |  |  |  |  |  |  |
| Height | X |  |  |  |  |  |  |  |  |  |  |  |  |  |
| Limited physical examination^§^ |  |  |  | X |  |  |  |  | X |  | X | X | X | X |
| Vital signs^║^ | X |  |  | X |  |  |  |  | X |  | X | X | X | X |
| ECG^¶^ | X |  |  |  |  |  |  |  | X |  |  | X | X |  |
| Serum pregnancy test | X |  |  |  |  |  |  |  |  |  |  |  |  |  |
| Urine pregnancy test |  |  |  | X |  | X | X | X |  |  | X |  | X |  |
| Haematology^#^ | X | X | X | X |  | X | X | X | X | X | X | X | X | X |
| Coagulation (PT/PTT/INR) | X |  |  |  |  |  |  |  |  |  |  |  |  |  |
| Chemistry** | X |  |  | X |  |  |  |  | X |  | X | X | X |  |
| Urinalysis | X |  |  |  |  |  |  |  |  |  |  |  |  |  |
| Serum periostin | X | X | X | X |  | X | X | X | X | X | X | X | X | X |
| PD markers (serum and plasma)^§§^ |  |  | X | X |  | X | X | X | X | X | X | X | X | X |
| Serum antibody sample^††^ |  |  |  | X |  |  |  |  | X |  | X | X | X | X^‡‡^ |
| Serum tryptase^║║^ |  |  |  |  | X^‡‡^ | | | | |  |  |  |  | X^‡‡^ |
| Spirometry | X | X | X | X |  | X | X | X | X | X | X | X | X | X |
| Sputum induction^¶¶^ |  |  | X |  |  |  |  |  |  |  |  |  |  |  |
| Bronchoscopy with brushings, biopsies^##^ |  |  |  | X |  |  |  |  | X |  |  |  |  |  |
| Whole blood RNA PAXgene^§§^ |  |  | X | X |  | X | X | X | X | X | X | X | X |  |
| Whole blood DNA (*GLCCl1* SNP analysis and optional PCR sample)^##^ |  |  |  | X |  |  |  |  |  |  |  |  |  |  |
| Transfer of In2itive e-diary data | X | X | X | X |  | X | X | X | X | X | X | X | X |  |
| Bronchodilator reversibility*** | X | X | X |  |  |  |  |  |  |  |  |  |  |  |
| FeNO^†††^ | X | X | X | X |  | X | X | X | X | X | X | X | X | X |
| ACQ-5^‡‡‡^ | X | X | X | X |  |  |  |  | X |  | X | X | X |  |
| AQLQ(S)^§§§^ |  |  |  | X |  |  | X | X | X | X | X | X | X | X |
| CXR^║║║^ | X |  |  |  |  |  |  |  |  |  |  |  |  |  |
| Randomisation |  |  |  |  | X |  |  |  |  |  |  |  |  |  |
| Study drug administration |  |  |  |  |  | X | X | X | X |  |  |  |  |  |
| Concomitant medications | X | X | X | X | X | X | X | X | X | X | X | X | X | X |
| Adverse events | X | X | X | X | X | X | X | X | X | X | X | X | X | X |
| Asthma exacerbation assessment |  |  |  |  |  | X | | | | | | | | |

ACQ-5, 5-Item Asthma Control Questionnaire; ALT, alanine aminotransferase; AQLQ(S), Standardized Asthma Quality of Life Questionnaire; AST, aspartate aminotransferase; ATA, antitherapeutic antibody; BUN, blood urea nitrogen; CT, computed tomography; CXR, chest x-ray; FeNO, fractional exhaled nitric oxide; FEV_1_, forced expiratory volume in 1 second; HEENT, head, eyes, ears, nose, throat; ICS, inhaled corticosteroid; IL, interleukin; INR, international normalised ratio; LDH, lactate dehydrogenase; PCR, polymerase chain reaction; PD, pharmacodynamic; PEF, peak expiratory flow; PK, pharmacokinetic; PRO, patient-reported outcome; PT, prothrombin time; PTT, partial thromboplastin time; RCR, revised common rule; SNP, single nucleotide polymorphism.

Unless otherwise indicated, all assessments and sampling were performed prior to study drug administration.

* Visit 4a should be scheduled 7 days after visit 3.

^†^ The time period between visits 4a and 4b may be 0 to 48 hours (i.e., visits 4a and 4b could be on the same day or ≤2 days apart) based on the investigator’s discretion. Following bronchoscopy at visit 4a, the investigator should ensure that the patient does not experience a significant bronchoscopy-related adverse event (e.g., exacerbation requiring steroids and/or hospitalisation) before proceeding to visit 4b for randomisation and administration of study drug.

^‡^ Medical history includes smoking status, current treatment, duration of asthma, history of malignancy (if applicable), courses of rescue corticosteroids, asthma exacerbation history, and hospital admissions for asthma-related requirements in the previous year.

^§^ HEENT, cardiovascular, respiratory, and dermatologic examinations.

^║^ Heart rate and systolic and diastolic blood pressure while the patient is in a seated position.

^¶^ ECGs must be performed prior to any scheduled spirometry measurements, vital sign measurements, or blood draws.

^#^ Haemoglobin, haematocrit, platelet count, red and white blood cell counts, and percent and absolute differential count (neutrophils, eosinophils, lymphocytes, monocytes, basophils, and other cells).

** Sodium, potassium, chloride, bicarbonate, glucose, BUN, creatinine, calcium, phosphorus, magnesium, total and direct bilirubin, total protein, albumin, ALT, AST, LDH, alkaline phosphatase, creatine phosphokinase, and uric acid.

^††^ Whenever possible, an additional sample for serum PK and antibody analysis should be taken in the event of an anaphylactic, anaphylactoid, or serious hypersensitivity reaction.

^‡‡^ To be performed only if the patient has experienced an acute onset of symptoms of an anaphylactic, anaphylactoid, or serious hypersensitivity reaction.

^§§^ IL-13−related and other asthma (Th2, Th1, and Th17) pathway–related biomarkers.

^║║^ Whenever possible, patients who experience an acute onset of symptoms of an anaphylactic, anaphylactoid, or serious hypersensitivity reaction should have a blood sample for total serum tryptase analysis collected 1 to 6 hours after the event. The tryptase sample will be collected and analysed per the site’s local laboratory practice.

^¶¶^ Patients must have postbronchodilator FEV_1_ ≥60% predicted at visit 3 in order to undergo sputum induction. Sputum induction should not be performed within 7 days prior to bronchoscopy.

^##^ Patients should not undergo bronchoscopy if they have received oral corticosteroids in the previous 4 weeks or are experiencing an acute asthma exacerbation event at the time of the scheduled visit 8 (week 12).

*** Whole blood DNA will be analysed for the *GLCCl1* SNP. Patients will also have the option to store their remaining DNA sample in the RCR upon signing a separate RCR Informed Consent Form.

^†††^ Patients who do not have documentation of protocol-defined criterion for reversibility within the 12 months prior to visit 1 are required to be assessed for reversibility at visit 1, 2, or 3. Patients who do not meet the protocol-defined criterion for reversibility at visit 1 or 2 may repeat the test at visit 2 or 3, as necessary.

^‡‡‡^ FeNO is to be measured in the clinic with a handheld NIOX MINO device.

^§§§^ PRO questionnaires (ACQ-5 and AQLQ[S]) must be self-administered at the investigational site prior to the completion of other non-PRO assessments and before the patient receives any disease status information or study drug during that visit.

^║║║^ A CXR must be performed or scheduled at visit 1 unless a CXR or CT scan was obtained within 12 months prior to visit 1 and is available for review by the investigator. The x-ray may be performed locally but must be reviewed by the investigator prior to bronchoscopy at visit 4a. If a CXR (or CT scan) is not available in the 12 months preceding visit 1 or cannot be completed during screening, the patient will not be eligible for the study.

**Table E2.** Overall quality of the sample selection per bronchoscopy

| **Sample type** | **Failed, n** | **Passed, n** | **Total, n** | **Overall sample quality, % passed** |
| --- | --- | --- | --- | --- |
| Epithelium | 46 | 70 | 116 | 60 |
| Lamina propria | 14 | 102 | 116 | 88 |

**Table E3**. Summary of secondary airway epithelial eosinophil efficacy endpoints from baseline to week 12 in the primary analysis population. Estimates are based on a linear model that used relative change from baseline as the response variable and included terms for treatment number of asthma exacerbations within 12 months of study entry and baseline asthma medications. Relative change was defined as the absolute change from baseline to week 12, divided by the value at baseline. Placebo, n=12; lebrikizumab, n=14. Placebo-corrected adjusted mean change is the difference in adjusted mean changes between the lebrikizumab and placebo groups.

| **Secondary efficacy endpoint** | **Placebo-corrected adjusted mean change (95% CI)** |
| --- | --- |
| Absolute change per mm^2^ of basement membrane, cells/mm^2^ | 20 (−44 to 87) |
| Relative change per mm^2^ of basement membrane, % | 58.9 (−37.5 to 152.5) |
| Absolute change per volume of lamina propria, cells/μL | 46 (−1474 to 2390) |
| Relative change per volume of lamina propria, % | 750.6 (−516.4 to 2019.1) |

**Table E**4. Characteristics by race. Non-normal continuous data are summarized as median [1^st^ quartile, 3^rd^ quartile]. Normal continuous data are summarized as mean ± standard deviation. The number of non-missing continuous values are in curly braces. Categorical data is summarized as the counts (and percent of total) of each level (as indicated in table margins).

|  | **White** | **Black or African American** | **Asian** | **Unknown** |
| --- | --- | --- | --- | --- |
| N | 40 | 19 | 2 | 3 |
| Age (years) | 44 +/- 14 {40} | 45 +/- 9.9 {19} | 43 +/- 7.1 {2} | 58 +/- 9.2 {3} |
| Sex [F/M](n/%) | 20(50%)/20(50%) | 8(42.1%)/11(57.9%) | 1(50%)/1(50%) | 0(0%)/3(100%) |
| BMI (frac{kg}{m^2}) | 29 +/- 4.9 {40} | 30 +/- 5.2 {19} | 24 +/- 4.5 {2} | 33 +/- 1.9 {2} |
| Smoking Status [Never/Prev](n/%) | 34(85%)/6(15%) | 15(78.9%)/4(21.1%) | 2(100%)/0(0%) | 1(33.3%)/2(66.7%) |
| Duration of asthma (years) | 29 [20,39] {40} | 29 [24,36] {19} | 36 [26,47] {2} | 41 [32,48] {3} |
| ICS Fluticasone Equiv Daily Dose (mug/day) | 837 +/- 422 {40} | 743 +/- 391 {19} | 1000 +/- 707 {2} | 1167 +/- 764 {3} |
| Num Asthma Exac in Past 12 Mon [0/geq1](emph{N}/%) | 27(73%)/10(27%) | 10(55.6%)/8(44.4%) | 1(50%)/1(50%) | 1(33.3%)/2(66.7%) |
| ACQ5 | 2.5 +/- 0.81 {40} | 2.6 +/- 0.78 {19} | 3.2 +/- 0.85 {2} | 3.3 +/- 0.14 {2} |
| FEV1 pre-BD | 2.3 +/- 0.71 {40} | 2.1 +/- 0.63 {19} | 2.2 +/- 0.76 {2} | 2.2 +/- 0.32 {3} |
| FEV1% predicted pre-BD | 64 +/- 11 {40} | 66 +/- 11 {19} | 69 +/- 4.3 {2} | 64 +/- 7 {3} |
| FEV1/FVC (%) ratio pre-BD | 62 +/- 9 {40} | 64 +/- 8.8 {19} | 69 +/- 9.5 {2} | 57 +/- 0.47 {3} |
| Serum IgE (IU/mL) | 198 [44,421] {40} | 133 [76,237] {17} | 602 [28,1177] {2} | 151 [112,430] {3} |
| Blood Eosinophils (#/uL) | 240 [175,400] {40} | 220 [110,410] {19} | 550 [440,660] {2} | 230 [150,350] {3} |
| FeNO (ppB) | 24 [14,36] {40} | 31 [18,46] {19} | 53 [30,76] {2} | 27 [21,48] {3} |
| Serum Periostin (ng/mL) | 46 [41,58] {40} | 47 [38,57] {19} | 66 [55,76] {2} | 54 [46,93] {3} |
| Airway Subepithelial Eosinophils (cells/mm^2) | 412 +/- 400 {32} | 210 +/- 177 {16} | 180 +/- NA {1} | 30 +/- 43 {2} |
| Airway Subepithelial Collagen Thickness (um) | 5.6 +/- 1.5 {21} | 6.3 +/- 2 {12} |  | 6.2 +/- NA {1} |

**REFERENCES**

1. Ochkur SI, Kim JD, Protheroe CA, Colbert D, Condjella RM, Bersoux S, Helmers RA, Moqbel R, Lacy P, Kelly EA, Jarjour NN, Kern R, Peters A, Schleimer RP, Furuta GT, Nair P, Lee JJ, Lee NA. A sensitive high throughput ELISA for human eosinophil peroxidase: a specific assay to quantify eosinophil degranulation from patient-derived sources. *J Immunol Methods* 2012: 384(1-2): 10-20.

2. Hsia CC, Hyde DM, Ochs M, Weibel ER, Structure AEJTFoQAoL. An official research policy statement of the American Thoracic Society/European Respiratory Society: standards for quantitative assessment of lung structure. *Am J Respir Crit Care Med* 2010: 181(4): 394-418.

3. Corren J, Lemanske RF, Hanania NA, Korenblat PE, Parsey MV, Arron JR, Harris JM, Scheerens H, Wu LC, Su Z, Mosesova S, Eisner MD, Bohen SP, Matthews JG. Lebrikizumab treatment in adults with asthma. *N Engl J Med* 2011: 365(12): 1088-1098.

4. Hanania NA, Noonan M, Corren J, Korenblat P, Zheng Y, Fischer SK, Cheu M, Putnam WS, Murray E, Scheerens H, Holweg CT, Maciuca R, Gray S, Doyle R, McClintock D, Olsson J, Matthews JG, Yen K. Lebrikizumab in moderate-to-severe asthma: pooled data from two randomised placebo-controlled studies. *Thorax* 2015: 70(8): 748-756.

5. Noonan M, Korenblat P, Mosesova S, Scheerens H, Arron JR, Zheng Y, Putnam WS, Parsey MV, Bohen SP, Matthews JG. Dose-ranging study of lebrikizumab in asthmatic patients not receiving inhaled steroids. *J Allergy Clin Immunol* 2013: 132(3): 567-574.e512.

6. Miller MR, Hankinson J, Brusasco V, Burgos F, Casaburi R, Coates A, Crapo R, Enright P, van der Grinten CP, Gustafsson P, Jensen R, Johnson DC, MacIntyre N, McKay R, Navajas D, Pedersen OF, Pellegrino R, Viegi G, Wanger J, Force AET. Standardisation of spirometry. *Eur Respir J* 2005: 26(2): 319-338.

7. Hankinson JL, Odencrantz JR, Fedan KB. Spirometric reference values from a sample of the general U.S. population. *Am J Respir Crit Care Med* 1999: 159(1): 179-187.

8. Woodruff PG, Innes AL. Quantitative morphology using bronchial biopsies. *Eur Respir Rev* 2006: 15(101): 157-161.

9. Protheroe C, Woodruff SA, de Petris G, Mukkada V, Ochkur SI, Janarthanan S, Lewis JC, Pasha S, Lunsford T, Harris L, Sharma VK, McGarry MP, Lee NA, Furuta GT, Lee JJ. A novel histologic scoring system to evaluate mucosal biopsies from patients with eosinophilic esophagitis. *Clin Gastroenterol Hepatol* 2009: 7(7): 749-755.e711.

10. Ferrando RE, Nyengaard JR, Hays SR, Fahy JV, Woodruff PG. Applying stereology to measure thickness of the basement membrane zone in bronchial biopsy specimens. *J Allergy Clin Immunol* 2003: 112(6): 1243-1245.

11. Ordonez CL, Khashayar R, Wong HH, Ferrando R, Wu R, Hyde DM, Hotchkiss JA, Zhang Y, Novikov A, Dolganov G, Fahy JV. Mild and moderate asthma is associated with airway goblet cell hyperplasia and abnormalities in mucin gene expression. *Am J Respir Crit Care Med* 2001: 163(2): 517-523.

12. Brey EM, Lalani Z, Johnston C, Wong M, McIntire LV, Duke PJ, Patrick CW, Jr. Automated selection of DAB-labeled tissue for immunohistochemical quantification. *J Histochem Cytochem* 2003: 51(5): 575-584.

13. Jia G, Erickson RW, Choy DF, Mosesova S, Wu LC, Solberg OD, Shikotra A, Carter R, Audusseau S, Hamid Q, Bradding P, Fahy JV, Woodruff PG, Harris JM, Arron JR, Bronchoscopic Exploratory Research Study of Biomarkers in Corticosteroid-refractory Asthma Study G. Periostin is a systemic biomarker of eosinophilic airway inflammation in asthmatic patients. *J Allergy Clin Immunol* 2012: 130(3): 647-654.e610.
